# Ruxolitinib binding to human serum albumin: bioinformatics, biochemical and functional characterization in JAK2V617F^+^ cell models

**DOI:** 10.1038/s41598-019-52852-9

**Published:** 2019-11-08

**Authors:** Elisabetta De Marinis, Alessia Ceccherelli, Alberto Quattrocchi, Loris Leboffe, Fabio Polticelli, Clara Nervi, Paolo Ascenzi

**Affiliations:** 1 Department of Medical and Surgical Sciences and Biotechnologies, Sapienza University of Rome, I-04100 Latina, Italy; 2 Department of Sciences, Roma Tre University, I-00146 Roma, Italy; 3 National Institute of Nuclear Physics, Roma Tre Section, I-00146 Roma, Italy; 4 Interdepartmental Laboratory for Electron Microscopy, Roma Tre University, I-00146 Roma, Italy

**Keywords:** Biochemistry, Molecular medicine

## Abstract

Ruxolitinib is a type I JAK inhibitor approved by FDA for targeted therapy of Philadelphia-negative myeloproliferative neoplasms (MPNs), all characterized by mutations activating the JAK2/STAT signaling pathway. Treatment with ruxolitinib improves constitutional symptoms and splenomegaly. However, patients can become resistant to treatment and chronic therapy has only a mild effect on molecular/pathologic remissions. Drugs interaction with plasma proteins, *i.e*. human serum albumin (HSA), is an important factor affecting the intensity and duration of their pharmacological actions. Here, the ruxolitinib recognition by the fatty acid binding sites (FAs) 1, 6, 7, and 9 of HSA has been investigated from the bioinformatics, biochemical and/or biological viewpoints. Docking simulations indicate that ruxolitinib binds to multiple sites of HSA. Ruxolitinib binds to the FA1 and FA7 sites of HSA with high affinity (*K*_r_ = 3.1 μM and 4.6 μM, respectively, at pH 7.3 and 37.0 °C). Moreover, HSA selectively blocks, in a dose dependent manner, the cytotoxic activity of ruxolitinib in JAK2V617F^+^ cellular models for MPN, *in vitro*. Furthermore this event is accompanied by changes in the cell cycle, p27^Kip1^ and cyclin D3 levels, and JAK/STAT signaling. Given the high plasma concentration of HSA, ruxolitinib trapping may be relevant *in vivo*.

## Introduction

Philadelphia-negative myeloproliferative neoplasms (MPNs), comprising polycythemia vera (PV), essential thrombocythemia (ET), and myelofibrosis (MF), are hematopoietic stem cell-derived disorders. These diseases are characterized by somatic driver mutations mainly occurring in Janus kinase 2 (JAK2), calreticulin (CALR) and thrombopoietin receptor (MPL) genes, all of them playing a role in driving the myeloproliferative phenotype^1^. These mutations activate the JAK2/signal transducer and activator of transcription (STAT) signaling pathway, which mediates the activity of various cytokine receptors involved in myelopoiesis, including the erythropoietin and thrombopoietin receptors^1–3^.

Most MPN patients (about 80%) display a single point mutation on the JAK2 gene, determining the Val617Phe mutation (*i.e*., JAK2V617F) within the pseudokinase domain. Through its enhanced, constitutive kinase activity, JAK2V617F deregulates myeloid cell proliferation, apoptosis and differentiation and was identified as a major cause of MPNs^2–4^. These insights led to the development of JAK inhibitors for the treatment of MPN patients and other diseases presenting de-regulated JAK/STAT signaling^5–7^.

Ruxolitinib (INCB018424, CAS 1092939-17-7) is an active and specific type I JAK2/JAK1 inhibitor approved by the Food and Drug Administration for the treatment of MF and hydroxyurea-resistant or -intolerant PV. By targeting the ATP-binding domain of kinases in their active conformation, ruxolitinib suppresses clonal MPN stem/progenitor cells, induces apoptotic cell death and inhibits interleukin-6 signaling^6,8,9^.

In clinical trials, treatment with ruxolitinib controls hematocrit in hydroxyurea-resistant or -intolerant PV patients, whereas in MF patients it produces a reduction of splenomegaly and improves symptoms and quality of life, prolonging overall survival compared to placebo and best available therapy^9–13^. Despite these clinical benefits, insufficient response or resistance to ruxolitinib has been reported in about 15% of patients and chronic therapy with this drug has only a mild effect on molecular and pathologic remissions^3,7^. Indeed, a number of combinations with other drugs, which can improve ruxolitinib effectiveness, overcome resistance and ameliorate toxic effects, have been tested with variable success in several clinical trials or are currently under evaluation^3,7,9^.

Human serum albumin (HSA), the most abundant plasma protein (~750 μM)^14^, is decreased by circulating inflammatory cytokines, such as interleukin-6 and TNF-α, which are commonly elevated in MFs. In turn, these cytokines are markedly decreased by treatment with ruxolitinib, while HSA levels are increased^15,16^. Recently, HSA level has been suggested as an independent indicator for prognosis in MF patients uncorrelated with cytogenetic or mutation profiles^17,18^. HSA and serum cholesterol levels were used to develop a new cachexia index further enhancing prognostication in MF^19^.

Pharmacokinetic data indicate that at clinically relevant concentrations ruxolitinib binds to plasma proteins, mostly HSA (approximately 97%) (DrugBank^20^; PubChem^21^) that may impair the drug efficacy. However, to our knowledge, no information is available on the relevance of HSA:ruxolitinib recognition in the biological and clinical response of JAK2V617F^+^ MPNs to this JAK2 inhibitor.

Several methodological approaches have been used to investigate ligand binding to HSA^22–26^. Here, ruxolitinib recognition by HSA has been investigated from the bioinformatics, biochemical and functional viewpoints. Heme-Fe(III), dansyl-sarcosine and dansyl-arginine have been used as specific probes of the fatty acid (FA) sites 1, 3–4 and 7 (*i.e*., FA1, FA3-FA4 and FA7)^26–28^. Ruxolitinib is predicted to bind to the fatty acid (FA) sites 1, 6, 7, and 9 (FA1, FA6, FA7, and FA9, respectively) of HSA. Binding of ruxolitinib to the FA1 and FA7 sites has also been experimentally confirmed. Of note, FA1 and FA7 sites represent two of the most relevant drug binding clefts of HSA^27–33^. Moreover, HSA selectively impairs ruxolitinib biological effects in JAK2V617F^+^ cellular models for MPN diseases *in vitro*, thus supporting the relevance of HSA levels for the therapeutic potential of this drug in MPNs.

## Results

### Docking simulations of ruxolitinib binding to HSA

Docking simulations of ruxolitinib binding to HSA predict that the binding affinity of the drug for the FA1, FA6, FA7, and FA9 sites is similar, values ranging between −7.1 and −8.0 kcal mol^−1^ (Table 1).

**Table 1:** Results of docking simulations of ruxolitinib binding to HSA.

Site	Free energy (kcal × mol^−1^)	Hydrophobic interactions	Hydrogen bonds
FA9	−8.0	His146, Lys190, Ala191, Ala194, Arg197, Glu425, Asn429, Lys432, Val456, Leu463	Asp108, Val455, Gln459
	−7.6	Asp108, His146, Lys190, Ala191, Ala194, Arg197, Glu425, Asn429, Lys432,Val456, Gln459, Leu463,	Asp108, Ser193, Tyr452
FA1	−7.8	Asp108, Asn109, Pro110, Arg145, His146, Pro147, Ser193, Arg197, Glu425	Lys190
	−7.6	Asn109, Pro110, Leu112, Arg145, His146, Pro147, Ser193, Ala194, Glu425	Asp108, Arg145
	−7.3	Asp108, Arg145, His146, Pro147, Tyr148, Lys190, Ser193, Ala194, Arg197, Glu425	Asp108
	−7.2	Leu115, Phe134, Lys137, Tyr138, Glu141, Ile142, Tyr161, Arg186	Tyr138, Tyr161
	−7.2	Leu115, Phe134, Lys137, Tyr138, Ile142, Tyr161, Arg186	Phe134, Tyr161
FA6	−7.7	Arg209, Lys212, Ala213, Val216, Ser232, Val235, Leu327, Asp324, Ala350, Glu354	Arg209, Asp324, Glu354
FA7	−7.1	Leu103, Glu100, Gln104, Arg197, Gln204, Lys205. Val462	Tyr148, Glu465

**Figure 1:**
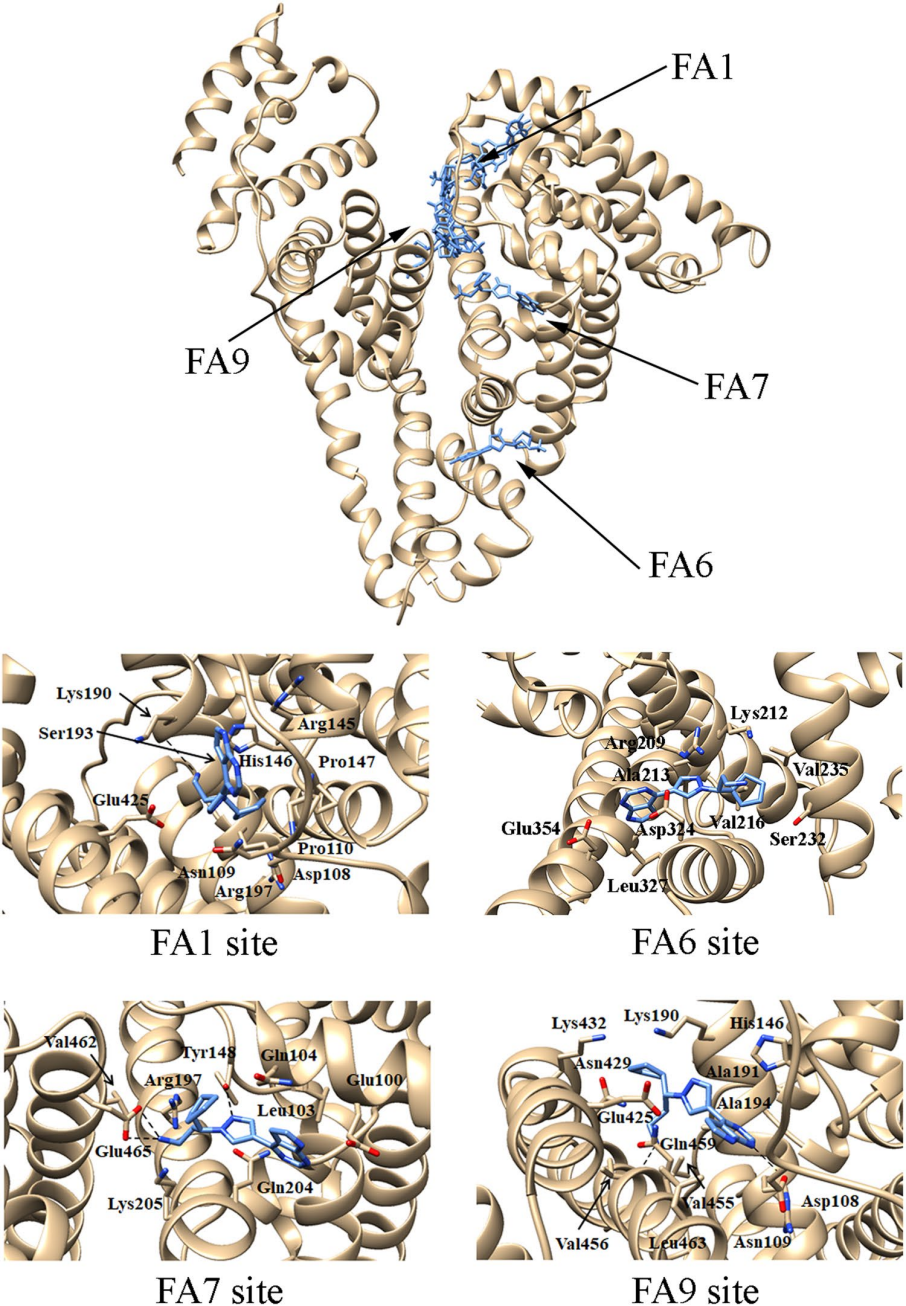
Ruxolitinib recognition mode by FA-free HSA as predicted by docking simulations. Top panel. Overall view of the nine lowest energy docking poses. Ruxolinitib poses are shown in stick representation and colored in blue. Bottom panels. Atomic details of ruxolitinib recognition at the FA1, FA6, FA7, and FA9 sites. The picture was drawn with the UCSF-Chimera v. 1.12 package^65^.

The preferential binding site of the drug is the FA9 cleft, located in the crevice between subdomains IA-IB-IIA on one side and subdomains IIB-IIIA-IIIB on the other side (Fig. 1 and Table 1). However, the number of complexes observed in the FA9 site in docking simulations with a maximum of 9 poses is only 2. The highest ranking complex of ruxolitinib bound to the FA9 site of FA-free HSA is shown in Fig. 1. In this site, the HSA:ruxolitinib complex is stabilized by hydrophobic interactions with Ala194, Ala191, Val456, and Leu463, and the aliphatic portions of His146, Lys190, Arg197, Glu425, Asn429, and Lys432. Moreover, the drug recognition is also based on the formation of hydrogen bonds with residues Asp108, Gln459, and the backbone amide group of Val455.

In other 5 poses, ruxolitinib is predicted to bind FA-free HSA at the FA1 site (*i.e*., at the subdomain IB), with a binding affinity value of the best ruxolitinib pose of −7.8 kcal × mol^−1^ (Table 1). Ruxolitinib forms hydrophobic interactions with Asp108, Asn109, Pro110, Arg145, His146, Pro147, Ser193, Arg197, and Glu425 and is hydrogen bonded to the backbone carbonyl group of Lys190 in the large FA1 site (Fig. 1). This pocket represents the third main ligand (*e.g*., drug) binding pocket of HSA^31,32^, the heme being a prototypical ligand^29,34^.

Ruxolitinib is also predicted to bind to FA6 site in the subdomain IIIA, the binding affinity value of the best pose being −7.7 kcal × mol^−1^ (Fig. 1 and Table 1). Ruxolitinib recognition by the FA6 site is based on the formation of hydrophobic interactions involving the drug and Arg209, Lys212, Ala213, Val216, Ser232, Val235, Leu327, Asp324, Ala350, and Glu354 residues. Moreover, ruxolitinib forms hydrogen bonds with the carboxyl group of Asp324 and Glu354, and with the amine group of Arg209 (Fig. 1).

Ruxolitinib binding to the FA7 site (also named Sudlow’s site I), which represents one of the most relevant drug binding site^29,35,36^, is stabilized by hydrophobic interactions with Leu103 and Val462 and the aliphatic portions of Glu100, Gln104, Arg197, Gln204, and Lys205. Moreover, the drug recognition is also based on the formation of hydrogen bonds with Tyr148 and Glu465 (Fig. 1 and Table 1).

### Thermodynamics of ruxolitinib binding to HSA

Since chromophores that bind selectively to the FA2, FA5, FA6, FA8, and FA9 sites are not available at present; only ruxolitinib binding to the FA1, FA3-FA4, and FA7 sites of HSA has been experimentally assessed.

**Figure 2:**
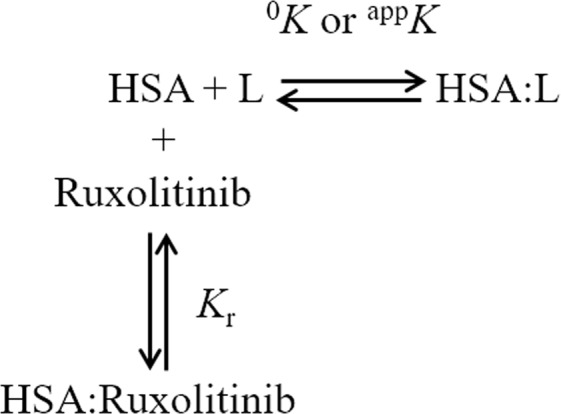
Competitive inhibition of heme-Fe(III), dansyl-arginine, and dansyl-sarcosine (i.e., L) binding to HSA by ruxolitinib. *K*_r_ indicates the dissociation equilibrium constant for ruxolitinib binding to HSA. ^0^*K* and ^app^*K* indicate the dissociation equilibrium constant for heme-Fe(III), dansyl-arginine and dansyl-sarcosine binding to HSA in the absence and presence of ruxolitinib, respectively.

**Figure 3:**
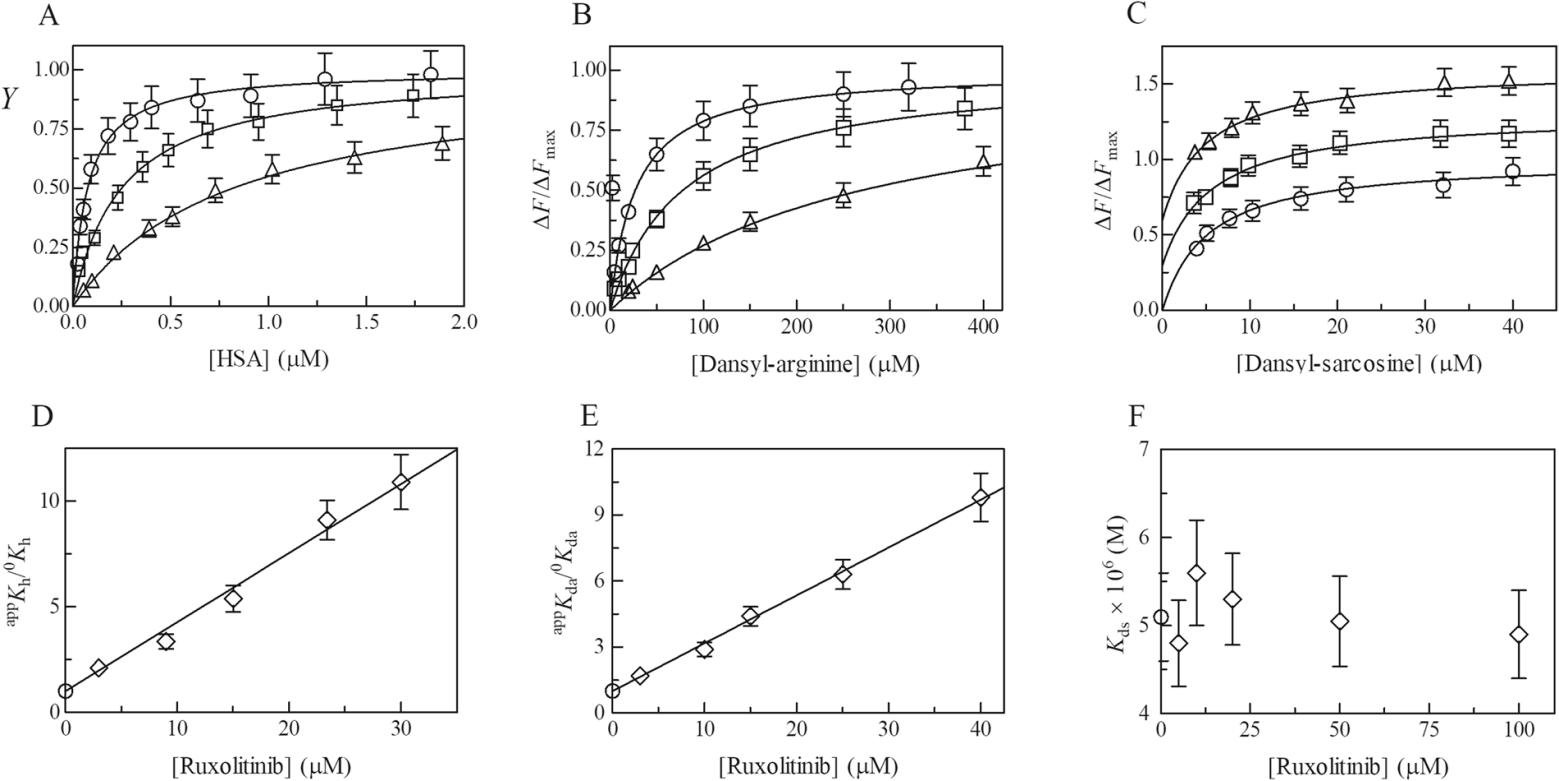
Effect of ruxolitinib on heme-Fe(III), dansyl-arginine, and dansyl-sarcosine binding to HSA, at pH 7.3 and 37.0 °C. (**A**) Binding isotherms for heme-Fe(III) binding to HSA in the absence (circles) and presence of 9.0 μM (squares) and 30 μM ruxolitinib (triangles). The heme-Fe(III) concentration was 1.3 μM. The HSA concentration refers to that of the free protein. The analysis of data according to Eq. (1) allowed the determination of the following parameters: ^0^*K*_h_ = 0.076 ± 0.008 μM (circles), ^app^*K*_h_ = 0.25 ± 0.03 μM (squares), and ^app^*K*_h_ = 0.82 ± 0.09 μM (triangles). (**B**) Binding isotherms for dansyl-arginine association to HSA in the absence (circles) and presence of 10 μM (squares) and 40 μM ruxolitinib (triangles) (panel A). The HSA concentration was 2.7 μM. The dansyl-arginine concentration refers to that of the free chromophore. The analysis of data according to Eq. (1) allowed the determination of the following parameters: ^0^*K*_da_ = 27 ± 3 μM (circles), ^app^*K*_da_ = 78 ± 8) μM (squares), and ^app^*K*_da_ = 27 ± 3 μM (triangles). (**C**) Binding isotherms for dansyl-sarcosine association to HSA in the absence (circles) and presence of 10 μM (squares) and 100 μM ruxolitinib (triangles) (panel A). The HSA concentration was 2.7 μM. The dansyl-sarcosine concentration refers to that of the free chromophore. For clarity, the binding isotherms for dansyl-sarcosine association to HSA in the presence of 10 μM and 100 μM ruxolitinib (diamonds and triangles, respectively) have been arbitrarily up shifted of 0.3 and 0.6 units. The analysis of data according to Eq. (2) allowed the determination of the following values parameters: ^0^*K*_ds_ = 5.1 ± 0.5 μM (circles), ^app^*K*_ds_ = 5.6 ± 0.6 μM (diamonds), and ^app^*K*_ds_ = 4.9 ± 0.5 μM (triangles). (**D**) Dependence of the ^app^*K*_h_/^0^*K*_h_ ratio for heme binding to HSA on the ruxolitinib concentration. The analysis of data according to Eq. (3) allowed the determination of the value of *K*_r = _3.1 ± 0.4 μM. The circle on the ordinate indicates the value of ^app^*K*_h_/^0^*K*_h_ = 1 obtained in the absence of ruxolitinib. (**E**) Dependence of the ^app^*K*_da_/^0^*K*_da_ ratio for dansyl-arginine binding to HSA on the ruxolitinib concentration. The analysis of data according to Eq. (3) allowed the determination of the value of *K*_r = _4.6 ± 0.5 μM. The circle on the ordinate indicates the value of ^app^*K*_da_/^0^*K*_da_ = 1 obtained in the absence of ruxolitinib. (**F**) Dependence of *K*_ds_ for dansyl-sarcosine on the ruxolitinib concentration. Values of ^app^*K*_ds_ are unaffected by ruxolitinib; in fact, the average value of ^app^*K*_ds_ 5.1 μM corresponds to that of ^0^*K*_ds_. *K*_h_*, K*_da_, and *K*_ds_ indicate the dissociation equilibrium constant for heme-Fe(III), dansyl-arginine and dansyl-sarcosine binding to HSA, respectively, in the absence and presence of ruxolitinib (*i.e*., ^0^*K*_h_, ^0^*K*_da_, and ^0^*K*_da_; and ^app^*K*_h_, ^app^*K*_ds_ and ^app^*K*_ds_, respectively). Where not shown, the standard deviation is smaller than the symbol. For details, see text.

Ruxolitinib recognition by the FA1, FA3-FA4, and FA7 sites of HSA has been investigated by competitive inhibition of heme-Fe(III), dansyl-sarcosine and dansyl-arginine binding, respectively, at pH 7.3 and 37.0 °C (Fig. 2). According to Eq. (1)^25,26,37^, data shown in Fig. 3 (panels A, B, and C) indicate that heme-Fe(III), dansyl-sarcosine and dansyl-arginine bind to HSA with a simple equilibrium, in the absence and presence of ruxolitinib. In fact, values of the Hill coefficient *n* are unaffected by ruxolitinib under all the experimental conditions, ranging between 0.99 ± 0.02 and 1.01 ± 0.02. Values of *K*_h_ and *K*_da_ increase from (7.6 ± 0.8) × 10^−8^ M and (2.7 ± 0.3) × 10^−5^ M, respectively, in the absence of ruxolitinib to (8.2 ± 0.9) × 10^−7^ M and (2.7 ± 0.3) × 10^−4^ M, respectively, in the presence of the drug (*i.e*., ^app^*K*_h_ > ^0^*K*_h_ and ^app^*K*_da_ > ^0^*K*_da_; Fig. 3, panels A and B). On the other hand, values of *K*_ds_ are unaffected by the ruxolitinib concentration (*i.e*., ^0^*K*_ds_ = ^app^*K*_ds_; Fig. 3, panel C). Of note, values of ^0^*K*_h_, ^0^*K*_da_ and ^0^*K*_ds_ determined here well agree with those reported in the literature^24–28,38–40^.

Data shown in Fig. 3 (panels D and E) indicate that ruxolitinib inhibits competitively heme-Fe(III) and dansyl-arginine binding to HSA. In fact, values of ^app^*K*/^0^*K* for heme-Fe(III) and dansyl-arginine binding to HSA increase linearly on the drug concentration (*i.e*., [ruxolitinib]). The linear analysis of data according to Eq. (3) discards the possibility of the linkage between the FA1 and FA7 sites for ruxolitinib binding to HSA (*i.e*., allosteric effects). The values of the dissociation equilibrium constant for ruxolitinib binding to HSA (*i.e*., *K*_r_ = 3.1 ± 0.4 μM and 4.6 ± 0.5 μM; at pH 7.3 and 37.0 °C), determined according to Eq. (3), correspond to the inverse of the slope of the straight lines shown in panels D and E of Fig. 3^41^. Data shown in Fig. 3 (panels D and E) indicate that ruxolitinib binds to the FA1 and FA7 sites of HSA with similar affinity (*i.e*., 3.1 ± 0.4 μM and 4.6 ± 0.5 μM, respectively). As predicted from docking simulations, ruxolitinib does not inhibit dansyl-sarcosine binding to the FA3-FA4 site of HSA over the whole concentration range explored (Fig. 3, panel F).

### Biological consequences of ruxolitinib binding to HSA in JAK2V617F^+^ myeloid cell lines

**Figure 4:**
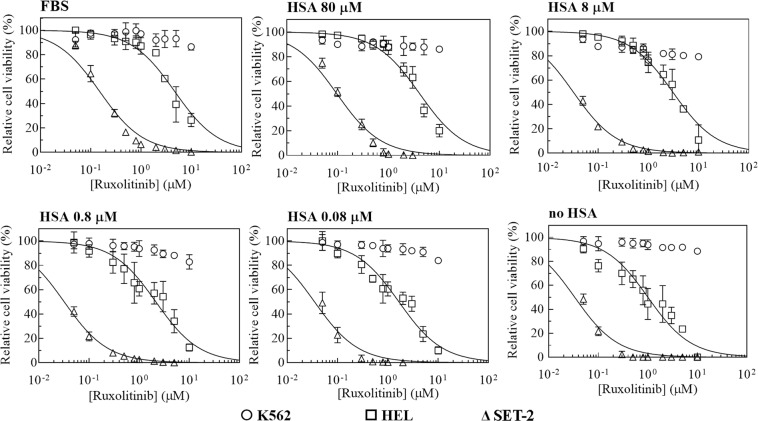
Effect of HSA levels on the in vitro sensitivity of JAK2wt K562 and JAK2V617F mutated HEL and SET-2 cells to ruxolitinib. JAK2wt K562 (circles) and JAK2V617F^+^ HEL (squares) and SET-2 (triangles) myeloid cell lines were cultured in 10% FBS medium or serum-free medium containing HSA (0–80 µM) and treated with the indicated doses of ruxolitinib for 72 hours. Cell viability was determined by the ATP-based cell-viability assay CellTiter-Glo. Data are the percent of untreated control cells. All data are presented as means of three independent experiments ± SD.

Patient-derived myeloid cell lines K562, HEL and SET-2, respectively carrying JAK2 wild type, homozygous and heterozygous JAK2V617F mutation^42^, were used to assess the ability of HSA to affect the ruxolitinib-mediated inhibition of cells viability. Cells were cultured in 10% FBS medium or serum-free medium containing HSA at concentrations ranging between 0.08 µM and 80 µM, the last one representing the concentration of α-fetoprotein, the fetal analog of serum albumin, in 10% FBS containing medium. Cells were treated with different doses of ruxolitinib for 72 hours (*i.e*., between 1.0 nM and 10 µM). In these culture conditions, HEL and SET-2 cells (both JAK2V617F positive *i.e*., JAK2V617F^+^) have been found to be sensitive to ruxolitinib, as previously demonstrated^8,43,44^ (Fig. 4 and Table 2). Nonetheless, HSA efficiently inhibits, in a concentration-dependent manner, the effect of ruxolitinib on cell viability (measured as *IC*_50_). In JAK2V617F^+^ HEL and SET-2 cells, the *IC*_50_ value of ruxolitinib respectively decreases from 4.7 ± 1.0 µM and 0.15 ± 0.04 µM, when seeded in 10% FBS or 80 µM HSA, to 1.0 ± 0.3 µM and 0.033 ± 0.008 µM, in the absence of HSA (Fig. 4 and Table 2). However, the *IC*_50_ value in JAK2wt K562 cells is greater than 10 µM under all the experimental conditions. On the basis of these results, we chose for the subsequent experiments the ruxolitinib concentrations that inhibit JAK2V617F^+^ cell viability of at least 30% (3 µM and 0.2 µM in HEL and SET-2 cells, respectively), while JAK2wt K562 cells were treated with 3 µM ruxolitinib.

**Table 2:** Effect of FBS and HSA on *IC*_50_ values for ruxolitinib-dependent K562, HEL and SET-2 cell viability.

*IC*_50_ (μM)						
Cell type	10% FBS	80 μM HSA	8 μM HSA	0.8 μM HSA	0.08 μM HSA	no HSA
K562	>10	>10	>10	>10	>10	>10
HEL	4.7 ± 1.0	4.1 ± 0.8	3.1 ± 0.6	2.2 ± 0.5	1.7 ± 0.4	1.0 ± 0.3
SET-2	0.15 ± 0.04	0.10 ± 0.03	0.033 ± 0.005	0.032 ± 0.004	0.035 ± 0.008	0.033 ± 0.008

**Figure 5:**
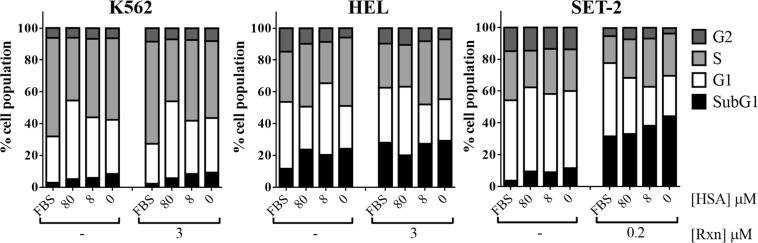
Effect of HSA levels on ruxolitinib-mediated changes in cell cycle distribution of JAK2wt K562 and JAK2V617F mutated HEL and SET-2 cells. Cell cycle analysis was performed in JAK2wt K562 and JAK2V617F^+^ HEL and SET-2 cells cultured in 10% FBS medium or serum-free medium containing 80 μM HSA, 8 μM HSA or medium alone (0 μM HSA) by propidium iodide staining followed by flow cytometry after 72 hours exposure to indicated concentrations of ruxolitinib (Rxn).

**Figure 6:**
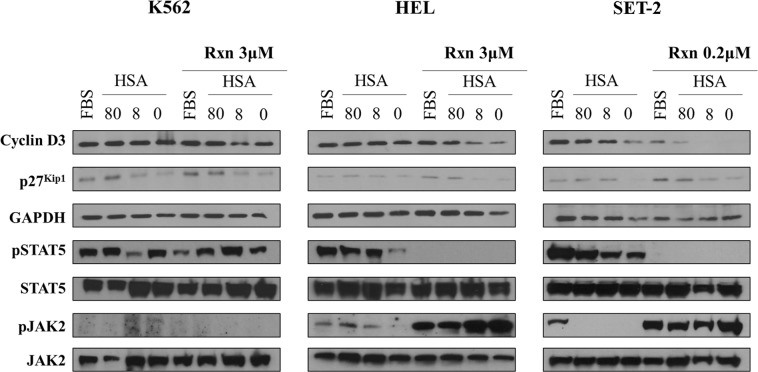
Effect of HSA levels on ruxolitinib-mediated changes of JAK2V617F signaling and cyclin expression levels. JAK2wt K562 and JAK2V617F^+^ HEL and SET-2 cells were cultured with FBS or 80 μM HSA, or 8 μM HSA, or medium alone (0 μM HSA). Cells were left untreated or treated with the indicated amount of ruxolitinib (Rxn). Change in the protein levels of inactive and phosphorylated STAT5 and JAK2 were measured after 3 hours of ruxolitinib treatment, whereas cyclin p27^Kip^, cyclin D3 and GAPDH protein levels were detected after 24 hours. Immunoblot analysis was performed as described in the method section.

FACS analysis of the cell cycle performed after 72 hours showed that, in the absence of ruxolitinib, the decrease of HSA concentrations in the medium slightly induces the sub-G1 phase in all the three myeloid cell lines (Fig. 5). Only in JAK2V617F^+^ HEL and SET-2 cells cultured at low HSA concentrations (8 µM) or in serum-free medium (no HSA), 72 hours treatment with ruxolitinib reduces the percentage of cells in the G1 phase and increases the percentage of cells in S phase, as compared to untreated cells (Fig. 5). Modification of cell cycle phases by HSA in ruxolitinib-treated JAK2V617F^+^ myeloid cells also corresponds to a change in the expression levels of both cyclin D3 and p27^Kip1^, two key regulators of the G1/S phase transition, as revealed after 24 hours by immunoblot analysis (Fig. 6). Overall these results suggest that HSA levels in growing medium selectively affect the ruxolitinib-mediated inhibition of cell viability and induce cell death in cells bearing the JAK2V617F mutation.

HSA affects also ruxolitinib-mediated effects on the JAK2/STAT5 signaling (Fig. 6). In the absence of ruxolitinib, HSA levels in culture medium influences, in a dose dependent manner, the phosphorylation status of both STAT5 and JAK2 in JAK2V617F^+^ HEL and SET-2 cells. These phosphorylation events are undetectable in JAK2wt K562 cells treated with 3 µM ruxolitinib and their specificity was further indicated by the lack of change in total STAT5 and JAK2 protein levels. As previously demonstrated for type I JAK inhibitors^45–47^, 3 hours treatment with ruxolitinib (at concentrations of 3 µM and 0.2 µM for HEL and SET-2, respectively) increases the activation-loop phosphorylation of JAK2, which coincides with the inhibition of STAT5 phosphorylation in JAK2V617F^+^ HEL and SET-2 cells, but not in JAK2wt K562 cells treated with 3 µM ruxolitinib. However, higher levels of phosphorylated JAK2 are induced by ruxolitinib in JAK2V617F^+^ cells grown at low HSA (8 µM) concentrations or in serum-free medium than in cells grown in medium containing 10% FBS or 80 µM HSA (Fig. 6). Overall, these results suggest that the ruxolitinib targeted inhibition of JAK2/STAT5 signaling is at least partially dependent on HSA serum levels, thus supporting a role for HSA in the therapeutic efficacy of ruxolitinib in MPNs.

## Discussion

Ruxolitinib is a clinically approved JAK1/JAK2 type I inhibitor specifically tailored to oncoproteins de-regulating JAK/STAT signaling. The positive, but not curative, effect of ruxolitinib in MPNs is well documented *in vitro* and *in vivo*. Overall, the toxic profile of ruxolitinib seems benign, with side effects observed in the patients, mainly of hematological type (thrombocytopenia and moderate anemia). Nevertheless, failure in the achievement of histopathological and molecular complete response or resistance to this drug represent important limitations to the use of ruxolitinib in MPN patients^3,7,9^. Various factors operating at the cellular or organismic level may be at the basis of these events. Ruxolitinib resistance can develop, for example, because of mutations of the kinase domain reported by *in vitro* studies^48,49^. However, these mutations have not been identified in patients yet, thus suggesting that ruxolitinib is a weak inhibitor^7^. Moreover, chronic exposure of MPN cells to ruxolitinib leads to disease persistence and reduced sensitivity to JAK inhibition^47^. In this context, ruxolitinib plasma concentration may represent an important concern for its therapeutic efficacy.

The interaction of drugs with plasma proteins, *i.e*. HSA, is an important factor affecting the duration and intensity of their pharmacological actions^14^. Here, we show that ruxolitinib is trapped by HSA impairing the HSA-based transport of physiological ligands (*e.g*., hemin) and the drug action. Given the *in vivo* concentrations of ruxolitinib and HSA (0.65 μM and 750 μM, respectively) (Fanali *et al*., 2012; DrugBank^20^; PubChem^21^) and the dissociation equilibrium constant for ruxolitib binding to HSA (*K*_r_ ranging between 3.1 and 4.6 μM; present study), for the chemical equilibrium laws we estimated that almost all ruxolitinib is bound to HSA *in vivo*. Indeed, it is known that approximately 97% of ruxolitinib is bound to plasma proteins, primarily to albumin (DrugBank^20^; PubChem^21^). Therefore, HSA has a big impact on the drug bioavailability.

In agreement with docking studies, ruxolitinib inhibits competitively heme-Fe(III) and dansyl-arginine binding to the FA1 and FA7 sites of HSA, respectively, without affecting dansyl-sarcosine association to the FA3-FA4 cleft, this being the binding site, among other ligands, of erucic acid^50^. Moreover docking investigations indicate that ruxolitinib binds to the FA9 and FA6 sites of HSA, however, no specific probes for these pockets are available to highlight drug recognition. Ruxolitinib binding to the FA1 cleft is predicted to occur in the vicinity of Tyr161, the HSA residue coordinating the heme-Fe(III) atom; this provides the molecular basis of the competitive inhibition exerted by ruxolitinib on heme-Fe(III) binding to HSA. Analogously, similar conclusions can be drawn for the competition between ruxolitinib and dansyl-arginine at the FA7 site.

High HSA concentration (80 µM) or 10% FBS in the growing medium renders myeloid cell lines derived from patients with JAK2V617F mutation more resistant to ruxolitinib. Indeed, the cytotoxic activity of ruxolitinib is enhanced if JAK2V617F^+^ HEL and SET-2 cells are grown in medium supplemented with low HSA concentrations (*i.e*. 8 and 0.08 µM HSA), and a maximal effect is reached in the absence of HSA or FBS (*i.e*., serum-free medium). We found that HSA affects ruxolitinib-mediated inhibition of cells viability HEL and SET-2 (both JAK2V617F^+^) in a dose-dependent manner and correlated with changes in the cell cycle distribution of these cells. In marked contrast, these events are not induced by HSA and/or ruxolitinib in JAK2wt K562 cells, thus indicating the importance of the JAK2V617F mutation in cell response.

At low HSA concentrations, treatment with ruxolitinib induced cell death signals as indicated by the progression of JAK2V617F^+^ HEL and SET-2 cells into the S phase of the cell cycle. The effect of HSA levels on ruxolitinib-mediated inhibition of cells viability is related also to changes in the JAK/STAT signaling, which is a characteristic feature of MPNs^46,51^. In JAK2V617F^+^ HEL and SET-2 myeloid cell lines grown at low HSA concentration, the ruxolitinib-induced activation-loop phosphorylation of JAK2 is increased and the tyrosine phosphorylation of STAT5 is inhibited. STAT5 is a known substrate of JAK2, whose activation is by itself sufficient to transform hematopoietic cells.

Overall, our findings suggest that ruxolitinib interaction with plasmatic HSA may be clinically relevant. Moreover, additional pathological conditions, the concurrent administration of drugs, age, and sex can affect drug (*e.g*., ruxolitinib) binding to plasma proteins^14,52–57^. In this view, modelling analysis of ruxolitinib recognition by HSA may be useful for the rational design of novel JAK2 inhibitors with higher therapeutic efficacy than the currently available drugs in MPNs. Moreover, therapeutic drug monitoring is demanding to determine the free-fraction of ruxolitinib, representing the drug fraction that has therapeutic efficacy.

## Materials

Fatty acid-free human serum albumin (HSA) was purchased from Kedrion (Lucca, Italy) (Albital 200 g/l, 20% solution) and Sigma-Aldrich (St. Louis, MO, USA). Ruxolitinib (INCB018424, INC424) was purchased from Selleckchem (Munich, Germany). Dansyl-arginine, dansyl-sarcosine, and hemin (Fe(III)-protoporphyrin IX) chloride were obtained from Sigma-Aldrich (St. Louis, MO, USA). All the other products were obtained from Merck AG (Darmstadt, Germany). All chemicals were of analytical or reagent grade and were used without further purification.

The HSA stock solution (1.0 × 10^−4^ M) was prepared by dissolving HSA in 2.0 × 10^−2^ M phosphate buffer, at pH 7.3. The HSA concentration was determined spectrophotometrically at 280 nm (ε = 3.82 × 10^4^ M^−1^ cm^−1^)^29^. The heme-Fe(III) stock solution (1.0 × 10^−3^ M) was prepared by dissolving heme-Fe(III) in 1.0 × 10^−2^ M NaOH^29^. The heme-Fe(III) concentration was determined spectrophotometrically at 535 nm, after converting heme-Fe(III) to the heme-Fe(III)-bis-imidazolate derivative by adding 1.0 M imidazole, in sodium dodecylsulfate micelles (ε = 1.45 × 10^4^ M^−1^ cm^−1^)^29^. Ruxolitinib was dissolved in DMSO whereas dansyl-arginine, and dansyl-sarcosine were dissolved in 2.0 × 10^−2^ M phosphate buffer, at pH 7.3^21,28^. The concentration of the ruxolitinib, dansyl-arginine, and dansyl-sarcosine stock solutions was 1.0 × 10^−3^ M, 1.0 × 10^−3^ M, and 4.0 × 10^−4^ M, respectively.

Human myeloid cell lines included JAK2V617F-mutated HEL and SET-2 cells, which are models for MPN diseases^42^, and JAK2wt K562 cells. HEL and K562 cells were grown in RPMI 1640 + GlutaMax medium (Gibco-BRL, Grand Island, USA), containing 10% heat-inactivated fetal bovine serum (FBS) (Gibco-BRL), 50 μg/ml streptomycin and 50 IU/ml penicillin. SET-2 cells were maintained in RPMI 1640 + GlutaMax medium (Gibco-BRL) supplemented with 20% heat-inactivated FBS (Gibco-BRL), 50 μg/ml streptomycin and 50 IU/ml penicillin. All cell lines were grown in a fully humidified incubator with 5% CO_2_ in air.

## Methods

### Docking simulations of ruxolitinib binding to HSA

The approach used to predict the molecular details of ruxolitinib binding to HSA is similar to that already used by us and other authors for other ligands^24–26,58,59^. In detail, docking simulations of ruxolitinib binding to HSA were performed using the crystal structure of ligand-free HSA (PDB ID: 1AO6)^60^. The three-dimensional structure of ruxolitinib was obtained from the crystal structure of C-Src in complex with ruxolitinib (PDB ID: 4U5J)^61^. Simulations were carried out using DockingApp^37^, a user friendly interface for the docking program AutoDock Vina^62^ which facilitates the setup, run and analysis of docking simulations. In order to carry out “blind” predictions of the ruxolitinib binding sites, in all the simulations the search space (docking grid) included the whole HSA structure. The grid spacing was set to 1 Å per grid unit and the exhaustiveness parameter was increased from the default value of 8 to 24, as suggested by AutoDock Vina developers for grid sizes larger than 27,000 Å^3^ ^62^, which is the case for HSA simulations. The simulations were carried out both by keeping all protein residues rigid and by allowing flexibility of only the residues building up the walls of the FA sites (FA1 to FA9). Residues for which flexibility was allowed are reported in Table S1 of Supplementary Materials. Rotatable bonds of ruxolitinib were kept flexible in the simulations.

### Thermodynamics of ruxolitinib binding to HSA

Thermodynamics of ruxolitinib recognition by the FA1, FA3-FA4 and FA7 sites of HSA was followed by competitive inhibition of heme-Fe(III), dansyl-sarcosine, and dansyl-arginine (*i.e*., L) binding, respectively, according to Fig. 1.

Heme-Fe(III) binding to HSA, in the absence and presence of ruxolitinib, was followed spectrophotometrically between 350 and 460 nm, at pH 7.3 (2.0 × 10^−2^ phosphate buffer) and 37.0 °C. The heme-Fe(III) concentration was 9.2 × 10^−7^ M, the ruxolitinib concentration ranged between 1.0 × 10^−7^ M and 1.0 × 10^−4^ M, and the HSA concentration ranged between 2.6 × 10^−7^ M and 1.9 × 10^−6^ M. Heme-Fe(III) binding to HSA, in the absence and presence of ruxolitinib, was investigated at a fixed heme-Fe(III) concentration by increasing the amount of HSA^24–26^.

Values of the dissociation equilibrium constant for heme-Fe(III) binding to HSA (*i.e*., *K*_h_), in the absence and presence of ruxolitinib, (*i.e*., ^0^*K*_h_ and ^app^*K*_h_, respectively; see Fig. 2) have been obtained from the dependence of the relative absorbance change (*i.e*., Δ*A*/Δ*A*_max_) of HSA:heme-Fe(III) complex formation on the free HSA concentration (*i.e*., [HSA]), according to Eq. (1)^24–26^:

1 $$\Delta A/\Delta {A}_{{\rm{\max }}}={[{\rm{HSA}}]}^{n}/({{K}_{{\rm{h}}}}^{n}+{[{\rm{HSA}}]}^{n})$$

where Δ*A* is the absorbance intensity change observed at each HSA concentration, Δ*A*_max_ is the maximum absorbance intensity change, and *n* is the Hill coefficient.

Dansyl-arginine and dansyl-sarcosine binding to HSA, in the absence and presence of ruxolitinib, was followed spectrofluorimetrically at pH 7.3 (2.0 × 10^−2^ M phosphate buffer) and 37.0 °C. The fluorophore of dansyl-arginine and dansyl-sarcosine was excited at 370 nm and the fluorescence emission intensities were measured at the maximum wavelengths (*i.e*., 460 nm for dansyl-arginine, and at 475 nm for dansyl-sarcosine); the excitation and emission slits were 5 nm^24–28,38–40^. The HSA concentration was 2.7 × 10^−6^ M, the dansyl-arginine and dansyl-sarcosine concentration ranged between 4.0 × 10^−6^ M and 1.5 × 10^−4^ M, and the ruxolitinib concentration ranged between 1.0 × 10^−7^ M and 1.0 × 10^−4^ M.

Values of the dissociation equilibrium constants for dansyl-arginine and dansyl-sarcosine binding to HSA (*i.e*., *K*_da_ and *K*_ds_, respectively), in the absence and presence of ruxolitinib, (*i.e*., ^0^*K*_da_ and ^app^*K*_da_, and ^0^*K*_ds_ and ^app^*K*_ds_ respectively; see Fig. 2) were obtained from the dependence of the relative fluorescence intensity change (*i.e*., Δ*F*/Δ*F*_max_) of the HSA:dansyl-arginine and HSA:dansyl-sarcosine complexes on the dansylated compound concentration in the absence (*i.e*., ^0^*K*) and presence (*i.e*., ^app^*K*) of ruxolitinib, according to Eq. (2)^24–28,38–40^:

2 $$\Delta F/\Delta {F}_{{\rm{\max }}}={[{\rm{HSA}}]}^{n}/({K}^{n}+{[{\rm{HSA}}]}^{n})$$

where Δ*F* is the fluorescence intensity change observed at each concentration of either dansyl-arginine or dansyl-sarcosine, Δ*F*_max_ is the maximum fluorescence intensity change, *K* is either ^0^*K*_da_ or ^app^*K*_da_ or ^0^*K*_ds_ or ^app^*K*_ds_, and *n* is the Hill coefficient. Since Δ*F* values were normalized dividing them by Δ*F*_max_, no correction of the inner filter effect was required. In fact, according to literature^63,64^ Δ*F*/Δ*F*_max_ is equal to Δ*F*^corrected^/Δ*F*_max_^corrected^.

The incubation time of ruxolitinib/HSA/heme-Fe(III), ruxolitinib/HSA/dansyl-arginine, and ruxolitinib/HSA/dansyl-sarcosine ranged between 10 and 30 min. Test measurements performed after 2 h of ruxolitinib/HSA/heme-Fe(III), ruxolitinib/HSA/dansyl-arginine, and ruxolitinib/HSA/dansyl-sarcosine incubation excluded slow kinetic effects.

According to the competitive inhibition mechanism shown in Fig. 2, values of *K*_r_ were obtained from the linear dependence of ^app^*K*/^0^*K* values on the ruxolitinib concentration according to Eq. (3)^41^:

3 $${}^{{\rm{app}}}K/{}^{0}K=([{\rm{ruxolitinib}}]/{K}_{{\rm{r}}})+1$$

Spectrophotometric and spectrofluorimetric measurements were carried out with a Jasco V-560 spectrophotometer and a Jasco FP-6500 spectrofluorometer (Jasco International Co., Ltd., Tokyo, Japan), respectively.

### Cell viability

The viability of cells was determined using the ATP-based cell-viability assay CellTiter-Glo (Promega, Madison, WI, USA) according to the manufacturer’s instruction. Briefly, cells were washed three times with PBS before seeding, resuspended in RPMI 1640 + 10% FBS or in RPMI-1640 serum-free medium containing HSA at concentrations ranging from 0 to 80 μM. A total of 40,000 cells/well were seeded in 96 well plates and treated with ruxolitinib at different concentrations (ranging from 0 to 10 μM). After 72 hours, the CellTiter-Glo solution (100 µl/well) was added to the cells for 20 minutes at RT and the cell viability was measured with a Tecan Spark 20 M multimode microplate reader (Tecan, Männerdorf, Switzerland), in luminescence mode (integration time 1 sec/well).

*IC*_50_ values, *i.e*. the concentration of ruxolitinib that gives half-maximal response on cell viability, have been obtained from the dependence of the relative cell viability (*i.e*., α) on the ruxolitinib concentration (*i.e*., [ruxolitinib]), at different culture conditions, according to Eq. (4):

4 $$\alpha =100\,\mbox{--}\,(100\times {[{\rm{ruxolitinib}}]}^{n}/(I{{C}_{50}}^{n}+{[{\rm{ruxolitinib}}]}^{n}))$$

where α is the relative cell viability and *n* is the Hill coefficient.

### Cell Cycle and cell death analysis

Cells were fixed in 70% ethanol in phosphate-buffered saline, incubated with 50 μg/mL propidium iodide (Sigma-Aldrich) and 50 units/mL DNase-free RNase A (Sigma-Aldrich) and analyzed using a Epics XL Cytometer (Beckman Coulter). A minimum of 10,000 total events were acquired.

### Immunoblotting

Proteins were fractionated by electrophoresis, electroblotted to nitrocellulose membrane (PROTRAN, Whatman Dassel, Germany) and probed with antibodies against STAT5a/b (cat. n. #9363), JAK2 (clone D2E12), phosphorylated STAT5 (Tyr684, clone D47E7), phospho-JAK2 (Tyr1007/1008, clone C80C3), GAPDH (clone 14C10) (Cell Signaling Technology, Danvers, MA, USA), Cyclin D3 (sc-182) and p27^Kip1^ (sc-528) (Santa Cruz Biotechnology, Dallas, TX, USA). Immunoreactivity was determined using the ECL method (Amersham Biosciences, Little Chalfont, UK), according to manufacturer’s instruction. Full-length gels and blots are included in the Supplementary Materials (Figures S1–S9).

### Data analysis

All data were analyzed using the GraphPad Prism program, version 6.01 (GraphPad Software, La Jolla, CA, USA). The results are given as mean values of at least three experiments plus or minus the corresponding standard deviation.

## Supplementary information

Supplementary information

## Data Availability

Data are available upon request to the authors.

## References

[1] Spivak JL (2017). Myeloproliferative Neoplasms. N Engl J Med.

[2] Vainchenker W, Constantinescu SN (2013). JAK/STAT signaling in hematological malignancies. Oncogene.

[3] Leroy E, Constantinescu SN (2017). Rethinking JAK2 inhibition: towards novel strategies of more specific and versatile Janus kinase inhibition. Leukemia.

[4] James C (2005). A unique clonal JAK2 mutation leading to constitutive signalling causes polycythaemia vera. Nature.

[5] Mascarenhas JO, Cross NC, Mesa RA (2014). The future of JAK inhibition in myelofibrosis and beyond. Blood Rev.

[6] Bose P, Verstovsek S (2017). JAK2 inhibitors for myeloproliferative neoplasms: what is next?. Blood.

[7] Vainchenker W (2018). JAK inhibitors for the treatment of myeloproliferative neoplasms and other disorders. F1000Res.

[8] Quintas-Cardama A (2010). Preclinical characterization of the selective JAK1/2 inhibitor INCB018424: therapeutic implications for the treatment of myeloproliferative neoplasms. Blood.

[9] Passamonti F, Maffioli M (2018). The role of JAK2 inhibitors in MPNs 7 years after approval. Blood.

[10] Harrison C (2012). JAK inhibition with ruxolitinib versus best available therapy for myelofibrosis. N Engl J Med.

[11] Verstovsek S (2012). A double-blind, placebo-controlled trial of ruxolitinib for myelofibrosis. N Engl J Med.

[12] Verstovsek S (2017). Long-term survival in patients treated with ruxolitinib for myelofibrosis: COMFORT-I and -II pooled analyses. J Hematol Oncol.

[13] Vannucchi AM (2015). Ruxolitinib versus standard therapy for the treatment of polycythemia vera. N Engl J Med.

[14] Fanali G (2012). Human serum albumin: from bench to bedside. Mol Aspects Med.

[15] Verstovsek S (2010). Safety and efficacy of INCB018424, a JAK1 and JAK2 inhibitor, in myelofibrosis. N Engl J Med.

[16] Mesa RA (2015). Effects of Ruxolitinib Treatment on Metabolic and Nutritional Parameters in Patients With Myelofibrosis From COMFORT-I. Clinical Lymphoma Myeloma & Leukemia.

[17] Lucijanic M (2018). Combining information on C reactive protein and serum albumin into the Glasgow Prognostic Score strongly discriminates survival of myelofibrosis patients. Blood Cells Molecules and Diseases.

[18] Lucijanic M (2018). Assessing serum albumin concentration, lymphocyte count and prognostic nutritional index might improve prognostication in patients with myelofibrosis. Wiener Klinische Wochenschrift.

[19] Tefferi A (2018). Development of a prognostically relevant cachexia index in primary myelofibrosis using serum albumin and cholesterol levels. Blood Adv.

[20] Wishart DS (2018). DrugBank 5.0: a major update to the DrugBank database for 2018. Nucleic Acids Res.

[21] PubChem. *Ruxolitinib, CID* 25126798, <https://pubchem.ncbi.nlm.nih.gov/compound/Ruxolitinib#section=Top> (2019).

[22] Sharif-Barfeh Z (2017). Multi-spectroscopic and HPLC Studies of the Interaction Between Estradiol and Cyclophosphamide With Human Serum Albumin: Binary and Ternary Systems. Journal of Solution Chemistry.

[23] Zolfagharzadeh M, Pirouzi M, Asoodeh A, Saberi MR, Chamani J (2014). A comparison investigation of DNP-binding effects to HSA and HTF by spectroscopic and molecular modeling techniques. J Biomol Struct Dyn.

[24] Di Muzio E (2014). Imatinib binding to human serum albumin modulates heme association and reactivity. Arch Biochem Biophys.

[25] Di Muzio E (2016). All-trans-retinoic acid and retinol binding to the FA1 site of human serum albumin competitively inhibits heme-Fe(III) association. Arch Biochem Biophys.

[26] Polticelli Fabio, Leboffe Loris, Tortosa Valentina, Trezza Viviana, Fanali Gabriella, Fasano Mauro, Ascenzi Paolo (2017). Cantharidin inhibits competitively heme-Fe(III) binding to the FA1 site of human serum albumin. Journal of Molecular Recognition.

[27] Sudlow G, Birkett DJ, Wade DN (1976). Further characterization of specific drug binding sites on human serum albumin. Mol Pharmacol.

[28] Sudlow G, Birkett DJ, Wade DN (1975). The characterization of two specific drug binding sites on human serum albumin. Mol Pharmacol.

[29] Fanali G, Ascenzi P, Bernardi G, Fasano M (2012). Sequence analysis of serum albumins reveals the molecular evolution of ligand recognition properties. J Biomol Struct Dyn.

[30] Leboffe L, di Masi A, Polticelli F, Trezza V, Ascenzi P (2019). Structural basis of drug recognition by human serum albumin. Curr Med Chem.

[31] Zsila F, Subdomain IB (2013). is the third major drug binding region of human serum albumin: toward the three-sites model. Mol Pharm.

[32] Zsila F (2013). Circular dichroism spectroscopic detection of ligand binding induced subdomain IB specific structural adjustment of human serum albumin. J Phys Chem B.

[33] Rabbani G, Ahn SN (2019). Structure, enzymatic activities, glycation and therapeutic potential of human serum albumin: A natural cargo. International Journal of Biological Macromolecules.

[34] Zunszain PA, Ghuman J, Komatsu T, Tsuchida E, Curry S (2003). Crystal structural analysis of human serum albumin complexed with hemin and fatty acid. BMC Struct Biol.

[35] Ghuman J (2005). Structural basis of the drug-binding specificity of human serum albumin. J Mol Biol.

[36] Curry S, Mandelkow H, Brick P, Franks N (1998). Crystal structure of human serum albumin complexed with fatty acid reveals an asymmetric distribution of binding sites. Nat Struct Biol.

[37] Di Muzio E, Toti D, Polticelli F (2017). DockingApp: a user friendly interface for facilitated docking simulations with AutoDock Vina. J Comput Aided Mol Des.

[38] Nakajou K, Watanabe H, Kragh-Hansen U, Maruyama T, Otagiri M (2003). The effect of glycation on the structure, function and biological fate of human serum albumin as revealed by recombinant mutants. Biochim Biophys Acta.

[39] Ryan AJ, Ghuman J, Zunszain PA, Chung CW, Curry S (2011). Structural basis of binding of fluorescent, site-specific dansylated amino acids to human serum albumin. J Struct Biol.

[40] Fanali G, Fasano M, Ascenzi P, Zingg JM, Azzi A (2013). alpha-Tocopherol binding to human serum albumin. Biofactors.

[41] Ascenzi P, Ascenzi MG, Amiconi G (1987). Enzyme competitive inhibition, graphical determination of Ki and presentation of data in comparative studies. Biochem Mol Biol Edu.

[42] Quentmeier H, MacLeod RA, Zaborski M, Drexler HG (2006). JAK2 V617F tyrosine kinase mutation in cell lines derived from myeloproliferative disorders. Leukemia.

[43] Kollmann K (2015). MARIMO cells harbor a CALR mutation but are not dependent on JAK2/STAT5 signaling. Leukemia.

[44] Bogani C (2013). mTOR inhibitors alone and in combination with JAK2 inhibitors effectively inhibit cells of myeloproliferative neoplasms. PLoS One.

[45] Tvorogov D (2018). Accumulation of JAK activation loop phosphorylation is linked to type I JAK inhibitor withdrawal syndrome in myelofibrosis. Sci Adv..

[46] Andraos R (2012). Modulation of activation-loop phosphorylation by JAK inhibitors is binding mode dependent. Cancer Discov.

[47] Koppikar P (2012). Heterodimeric JAK-STAT activation as a mechanism of persistence to JAK2 inhibitor therapy. Nature.

[48] Deshpande A (2012). Kinase domain mutations confer resistance to novel inhibitors targeting JAK2V617F in myeloproliferative neoplasms. Leukemia.

[49] Hornakova T (2011). Oncogenic JAK1 and JAK2-activating mutations resistant to ATP-competitive inhibitors. Haematologica.

[50] Rabbani G (2017). Binding of erucic acid with human serum albumin using a spectroscopic and molecular docking study. International Journal of Biological Macromolecules.

[51] Walz C (2012). Essential role for Stat5a/b in myeloproliferative neoplasms induced by BCR-ABL1 and JAK2(V617F) in mice. Blood.

[52] Grandison MK, Boudinot FD (2000). Age-related changes in protein binding of drugs: implications for therapy. Clin Pharmacokinet.

[53] Turnheim K (2004). Drug therapy in the elderly. Exp Gerontol.

[54] Turnheim K (2003). When drug therapy gets old: pharmacokinetics and pharmacodynamics in the elderly. Exp Gerontol.

[55] Ascenzi P, Fasano M (2010). Allostery in a monomeric protein: the case of human serum albumin. Biophys Chem.

[56] Ascenzi P, Fasano M (2009). Serum heme-albumin: an allosteric protein. IUBMB Life.

[57] di Masi A, Trezza V, Leboffe L, Ascenzi P (2016). Human plasma lipocalins and serum albumin: Plasma alternative carriers?. J Control Release.

[58] Rabbani G (2017). Biophysical Study on the Interaction between Eperisone Hydrochloride and Human Serum Albumin Using Spectroscopic, Calorimetric, and Molecular Docking Analyses. Molecular Pharmaceutics.

[59] Rabbani G, Lee EJ, Ahmad K, Baig MH, Choi I (2018). Binding of Tolperisone Hydrochloride with Human Serum Albumin: Effects on the Conformation, Thermodynamics, and Activity of HSA. Molecular Pharmaceutics.

[60] Sugio S, Kashima A, Mochizuki S, Noda M, Kobayashi K (1999). Crystal structure of human serum albumin at 2.5 A resolution. Protein Eng.

[61] Duan Y, Chen L, Chen Y, Fan XG (2014). c-Src binds to the cancer drug Ruxolitinib with an active conformation. PLoS One.

[62] Trott O, Olson AJ (2010). AutoDock Vina: improving the speed and accuracy of docking with a new scoring function, efficient optimization, and multithreading. J Comput Chem.

[63] Shakibapour N, Sani FD, Beigoli S, Sadeghian H, Chamani J (2019). Multi-spectroscopic and molecular modeling studies to reveal the interaction between propyl acridone and calf thymus DNA in the presence of histone H1: binary and ternary approaches. Journal of Biomolecular Structure & Dynamics.

[64] Sani FD (2018). Changes in binding affinity between ofloxacin and calf thymus DNA in the presence of histone H1: Spectroscopic and molecular modeling investigations. Journal of Luminescence.

[65] Pettersen EF (2004). UCSF Chimera–a visualization system for exploratory research and analysis. J Comput Chem.

