# Supplementary material for: Ruxolitinib binding to human serum albumin: bioinformatics, biochemical and functional characterization in JAK2V617F+ cell models
Source: Sci Rep. 2019 Nov 8;9:16379. doi: 10.1038/s41598-019-52852-9 (PMC6841977; doi:10.1038/s41598-019-52852-9)
Supplement: Supplementary file 1 — Supplementary information [file 41598_2019_52852_MOESM1_ESM.docx]

**Ruxolitinib binding to human serum albumin: bioinformatics, biochemical and functional characterization in JAK2V617F+ cell models**

Elisabetta De Marinis, Alessia Ceccherelli, Alberto Quattrocchi, Loris Leboffe, Fabio Polticelli, Clara Nervi, Paolo Ascenzi

**Supplementary Information data:**

**Supplementary figure S1-S9**: full lenght blots used in the figure 6 of the manuscript. The proteins detected by different antibodies and their molecular weights are indicated by the arrows.

**Table S1.** Residues for which flexibility was allowed in docking simulations of ruxolitinib binding to ligand-free HSA.


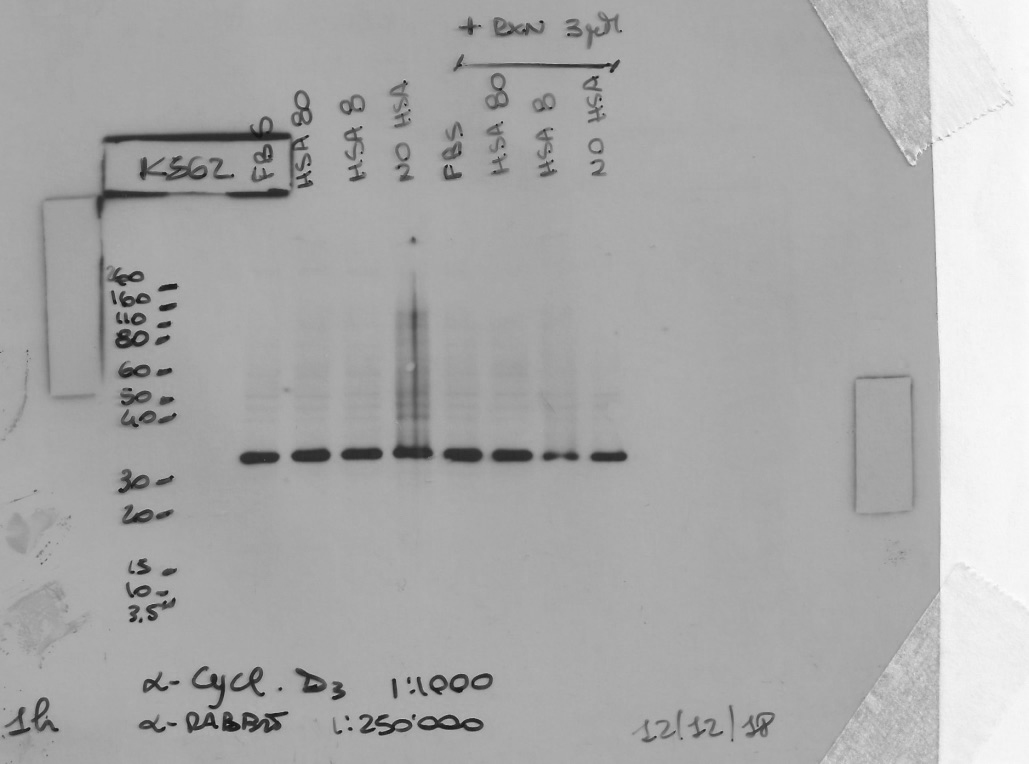


**K562 cells**

Molecular weight ladder (kDa)

α-Cyclin D3 (33 kDa)


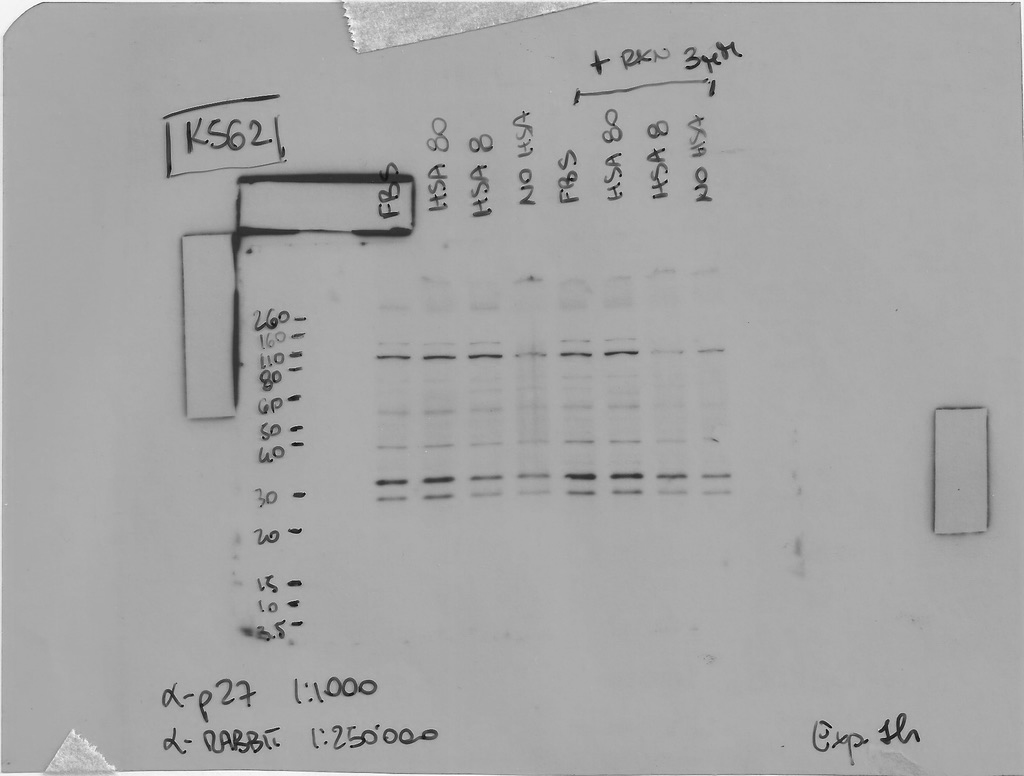


Molecular weight ladder (kDa)

α-p27 (27 kDa)


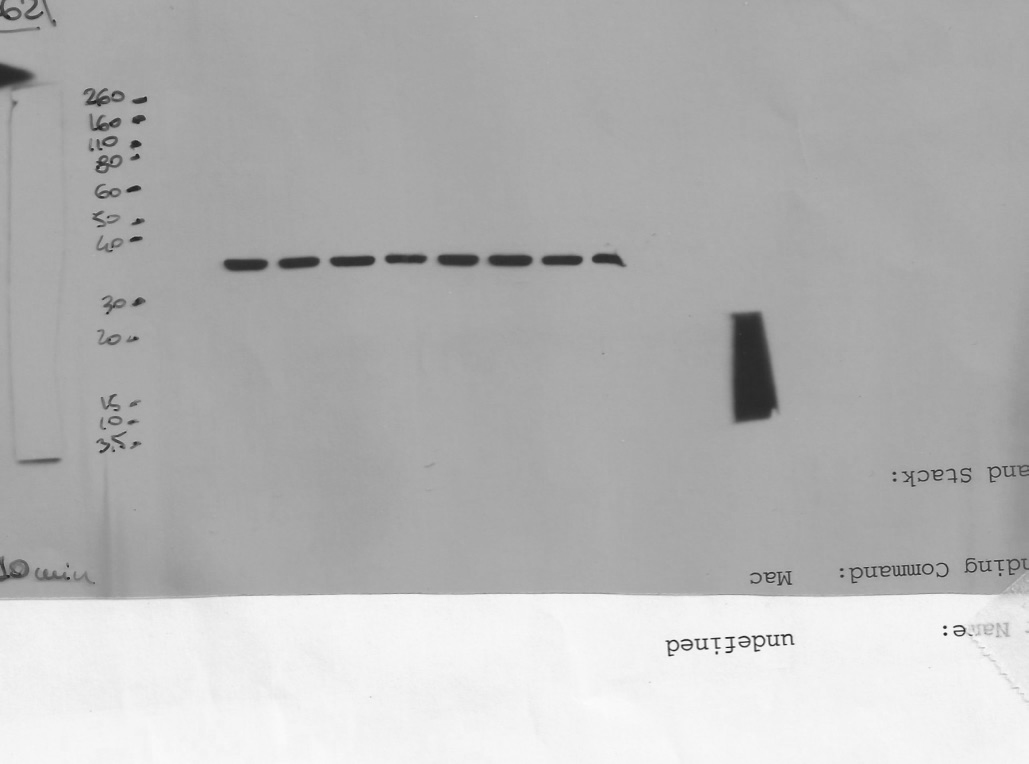


1

2

3

4

5

6

7

8

Molecular weight ladder (kDa)

α -GAPDH (37 kDa)

1. FBS
2. HSA 80 μM
3. HSA 8 μM
4. HSA 0 μM
5. FBS + Rxn 3 μM
6. HSA 80 μM + Rxn 3 μM
7. HSA 8 μM + Rxn 3 μM
8. HSA 0 μM + Rxn 3 μM

**S1**

Molecular weight ladder (kDa)


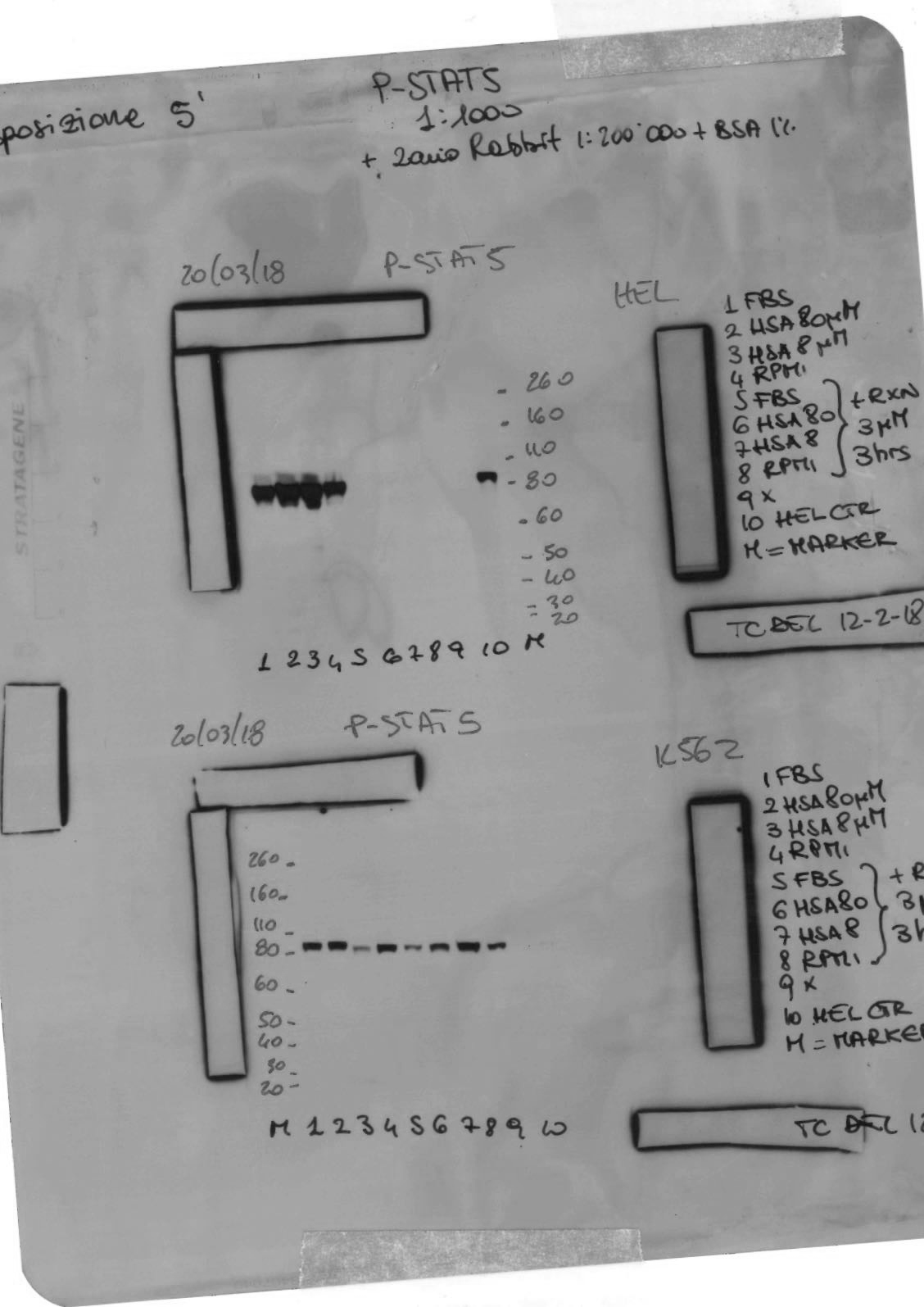


Molecular weight ladder (kDa)

α -phospho-STAT5 (90 kDa)

α -STAT5 (90 kDa)

1. FBS
2. HSA 80 μM
3. HSA 8 μM
4. HSA 0 μM
5. FBS + Rxn 3 μM
6. HSA 80 μM + Rxn 3 μM
7. HSA 8 μM + Rxn 3 μM
8. HSA 0 μM + Rxn 3 μM
9. Internal Control

1

2

3

4

5

6

7

8

9

**S2**

**K562 cells**

Molecular weight ladder (kDa)

Molecular weight ladder (kDa)

α -phospho-JAK2 (125 kDa)

α -JAK2 (125 kDa)

1. FBS
2. HSA 80 μM
3. HSA 8 μM
4. HSA 0 μM
5. FBS + Rxn 3 μM
6. HSA 80 μM + Rxn 3 μM
7. HSA 8 μM + Rxn 3 μM
8. HSA 0 μM + Rxn 3 μM
9. Internal Control

1

2

3

4

5

6

7

8

9


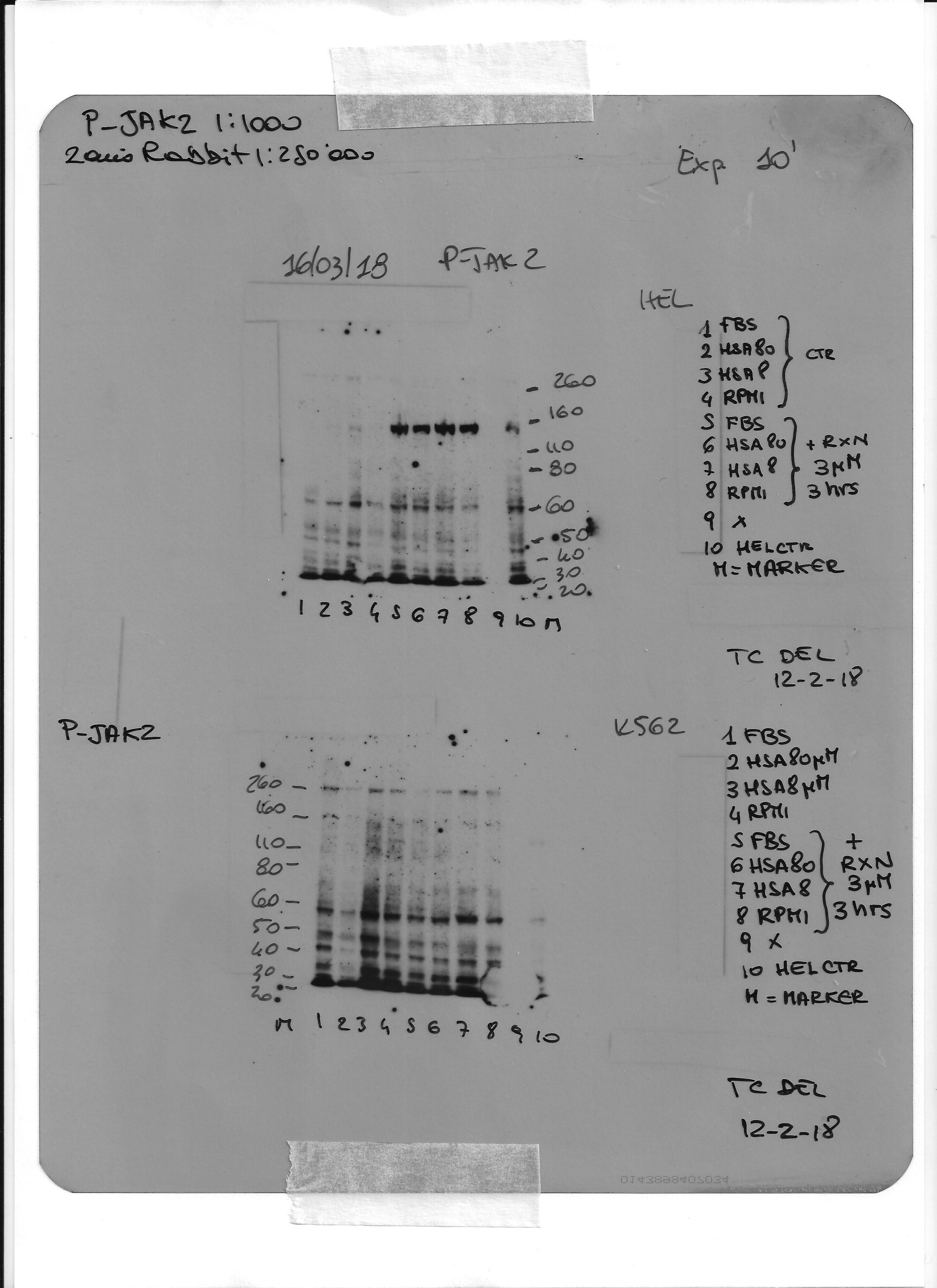

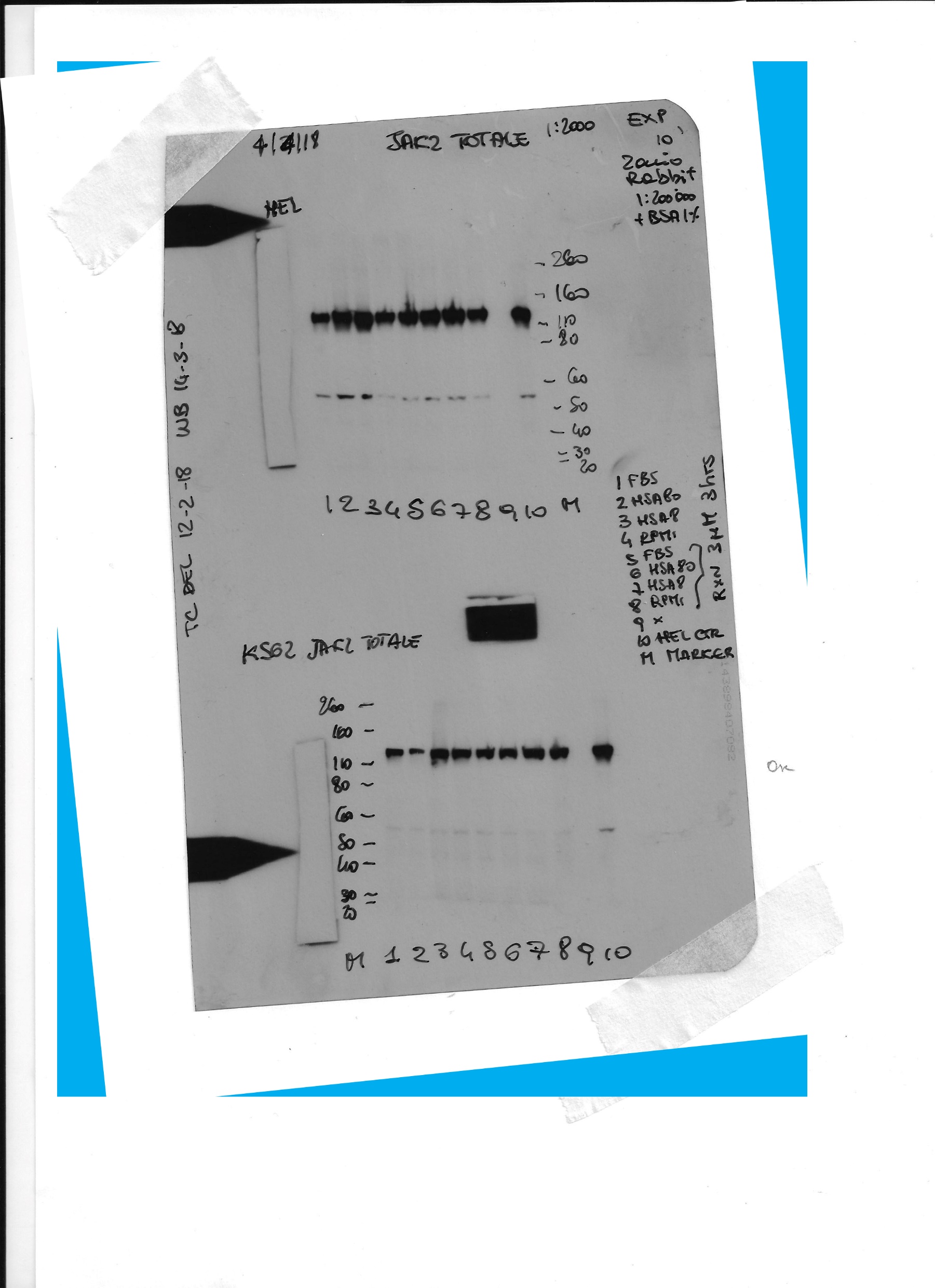


**S3**

**K562 cells**

**HEL cells**

Molecular weight ladder (kDa)

α -Cyclin D3 (33 kDa)

Molecular weight ladder (kDa)

α -p27 (27 kDa)

1

2

3

4

5

6

7

8

Molecular weight ladder (kDa)

α -GAPDH (37 kDa)

1. FBS
2. HSA 80 μM
3. HSA 8 μM
4. HSA 0 μM
5. FBS + Rxn 3 μM
6. HSA 80 μM + Rxn 3 μM
7. HSA 8 μM + Rxn 3 μM
8. HSA 0 μM + Rxn 3 μM


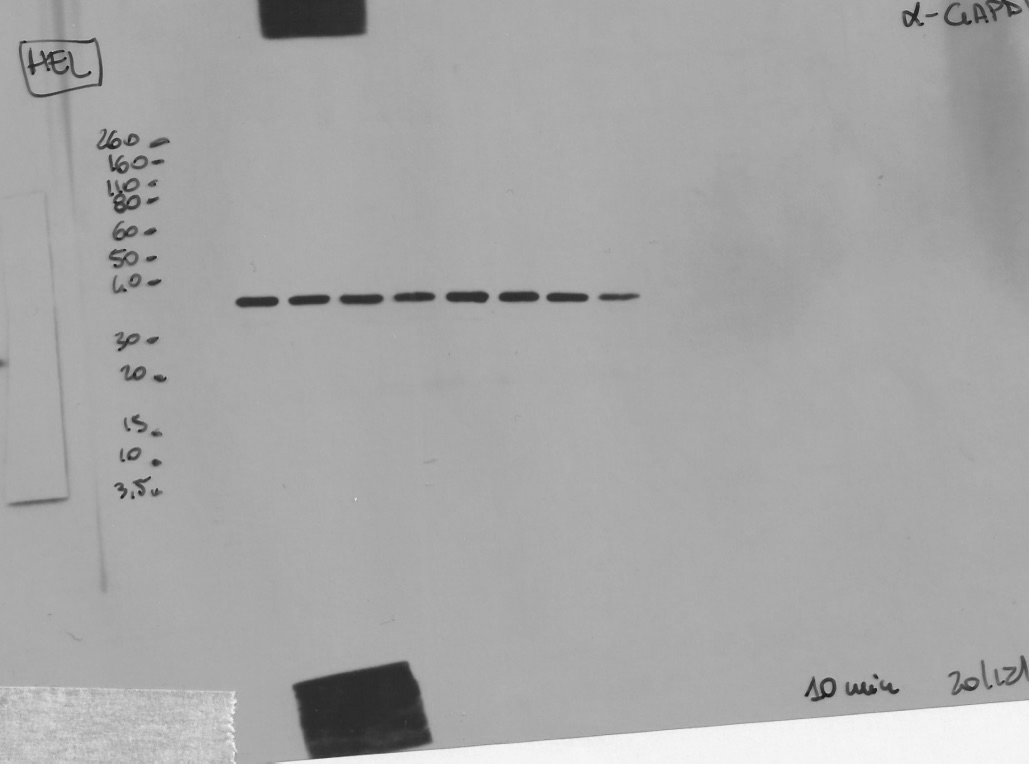

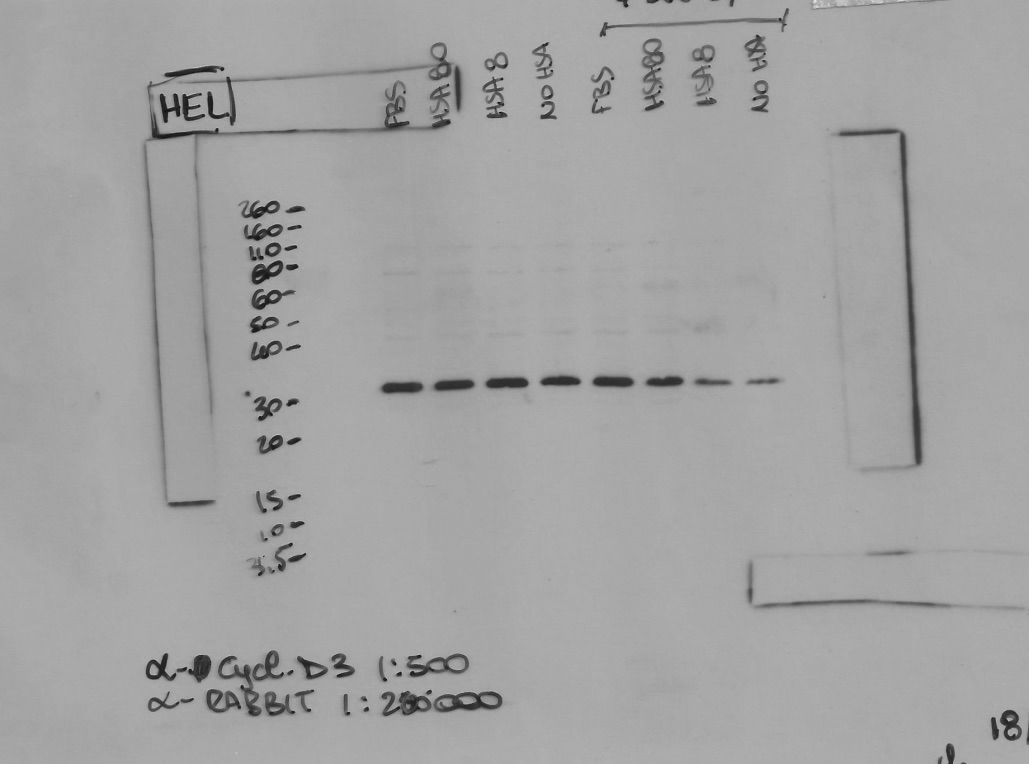

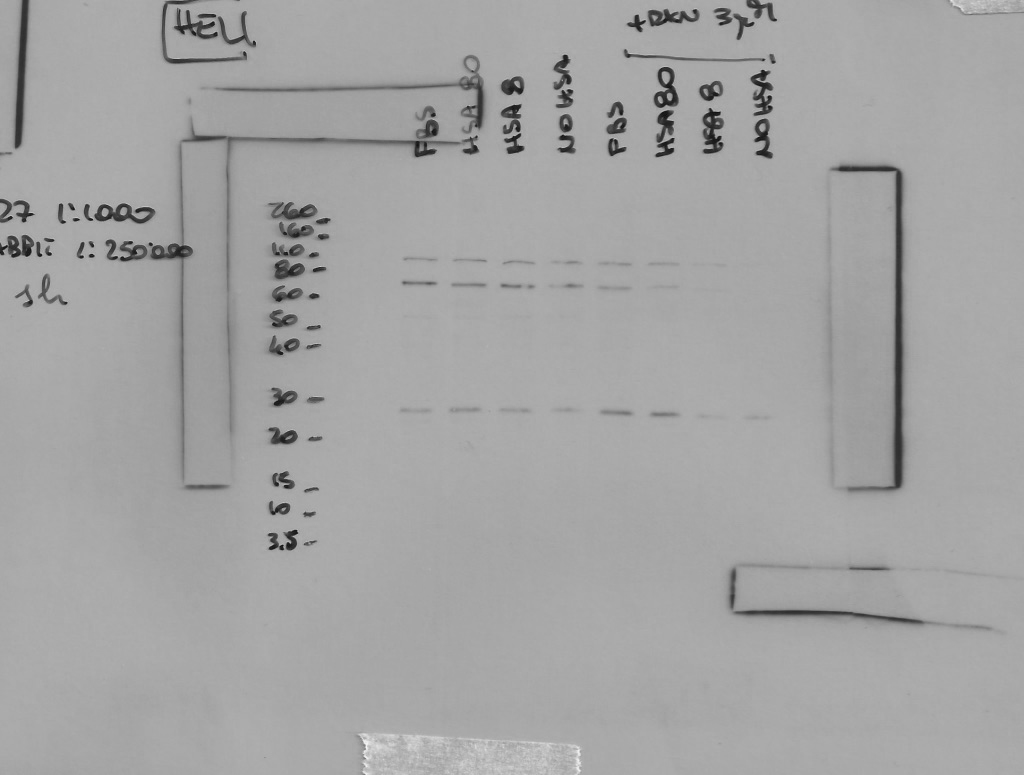


**S4**

Molecular weight ladder (kDa)

Molecular weight ladder (kDa)

α -phospho-STAT5 (90 kDa)

1. FBS
2. HSA 80 μM
3. HSA 8 μM
4. HSA 0 μM
5. FBS + Rxn 3 μM
6. HSA 80 μM + Rxn 3 μM
7. HSA 8 μM + Rxn 3 μM
8. HSA 0 μM + Rxn 3 μM
9. Internal Control

1

2

3

4

5

6

7

8

9


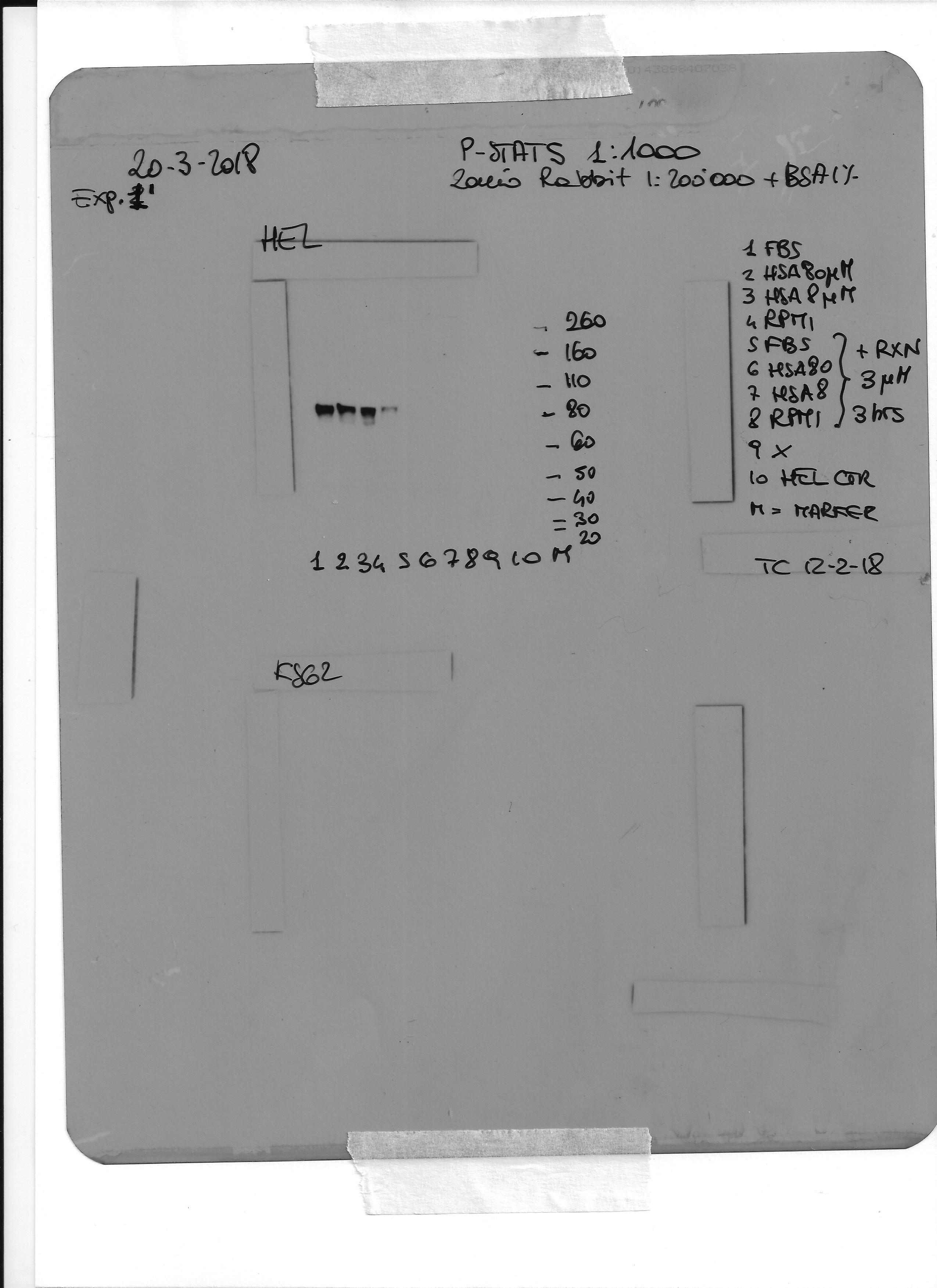

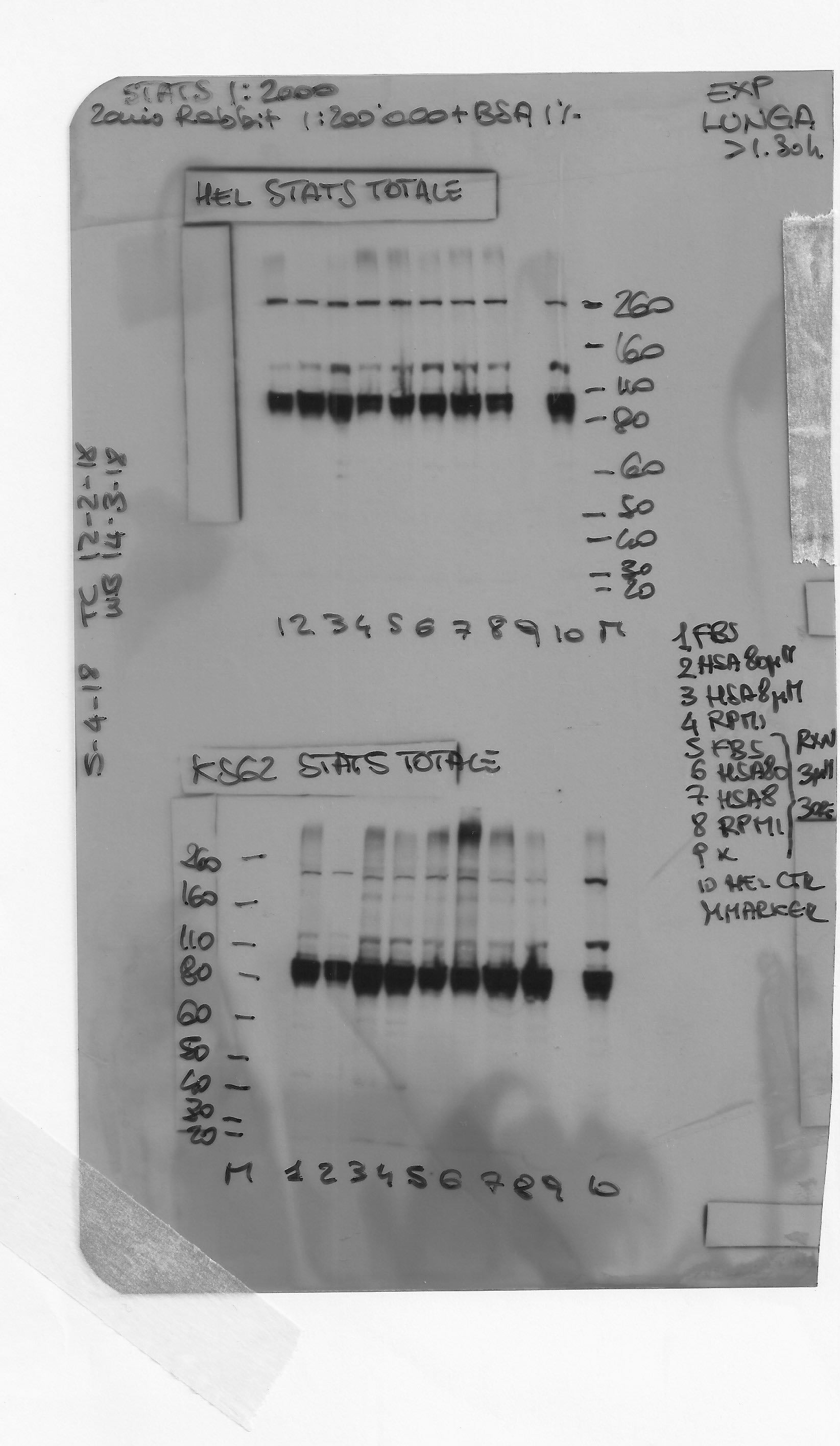


α -STAT5 (90 kDa)

**S5**

**HEL cells**

Molecular weight ladder (kDa)

Molecular weight ladder (kDa)

1. FBS
2. HSA 80 μM
3. HSA 8 μM
4. HSA 0 μM
5. FBS + Rxn 3 μM
6. HSA 80 μM + Rxn 3 μM
7. HSA 8 μM + Rxn 3 μM
8. HSA 0 μM + Rxn 3 μM
9. Internal Control

1

2

3

4

5

6

7

8

9


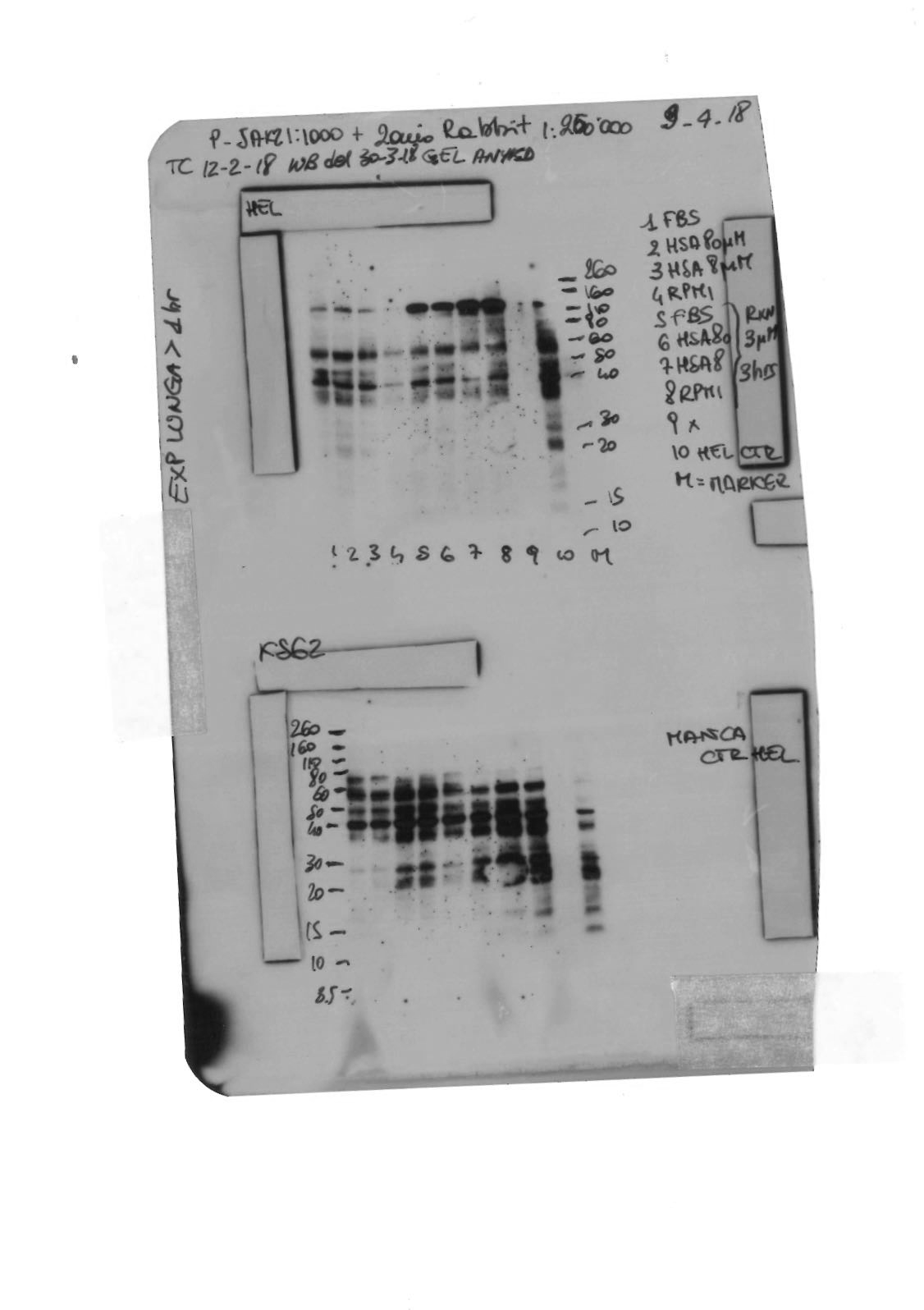

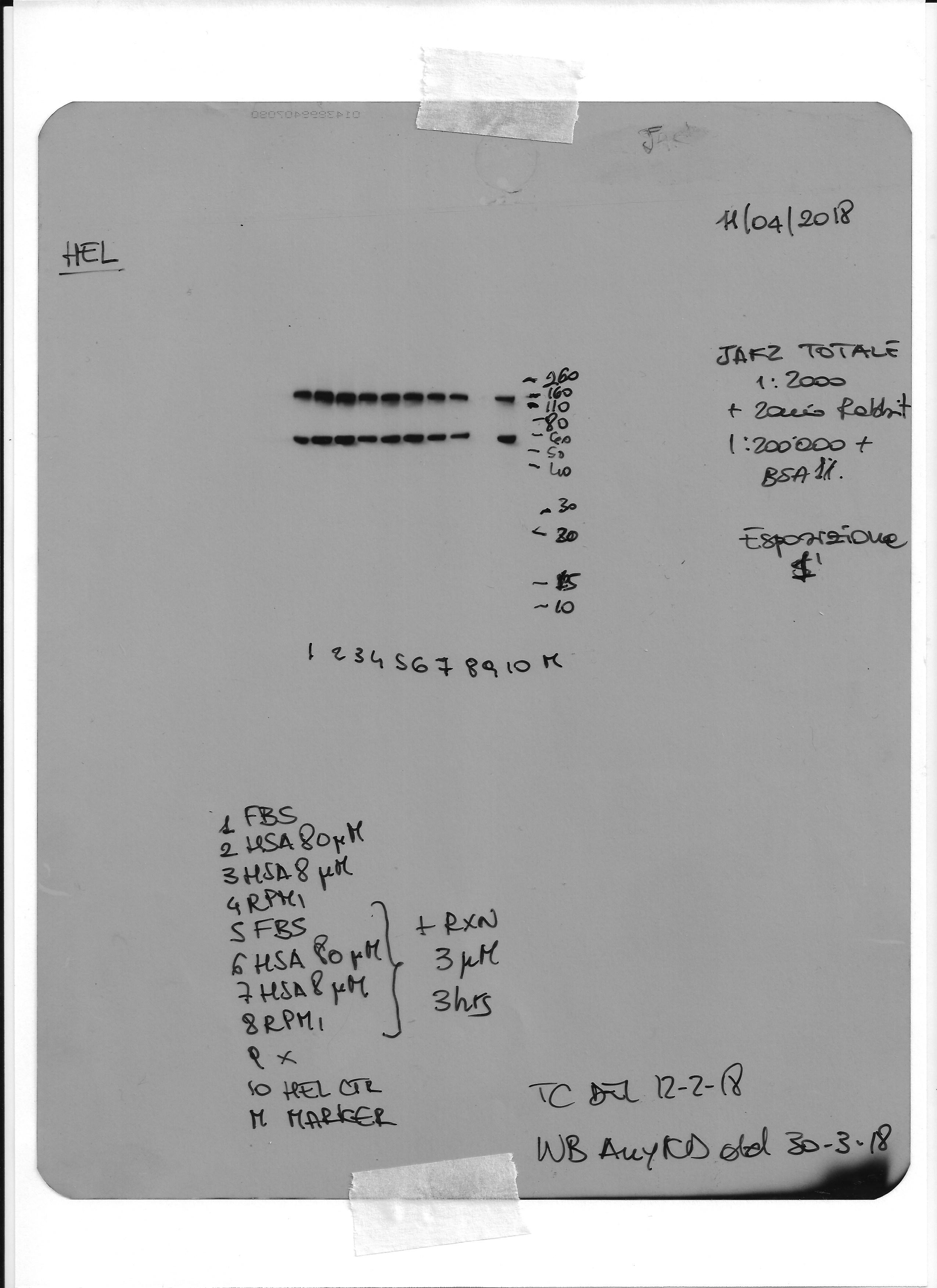


α –phospho-JAK2 (125 kDa)

α -JAK2 (125 kDa)

**S6**

**HEL cells**

**SET2 cells**

Molecular weight ladder (kDa)

Molecular weight ladder (kDa)

1

2

3

4

5

6

7

8

Molecular weight ladder (kDa)

1. FBS
2. HSA 80 μM
3. HSA 8 μM
4. HSA 0 μM
5. FBS + Rxn 0.2 μM
6. HSA 80 μM + Rxn 0.2 μM
7. HSA 8 μM + Rxn 0.2 μM
8. HSA 0 μM + Rxn 0.2 μM


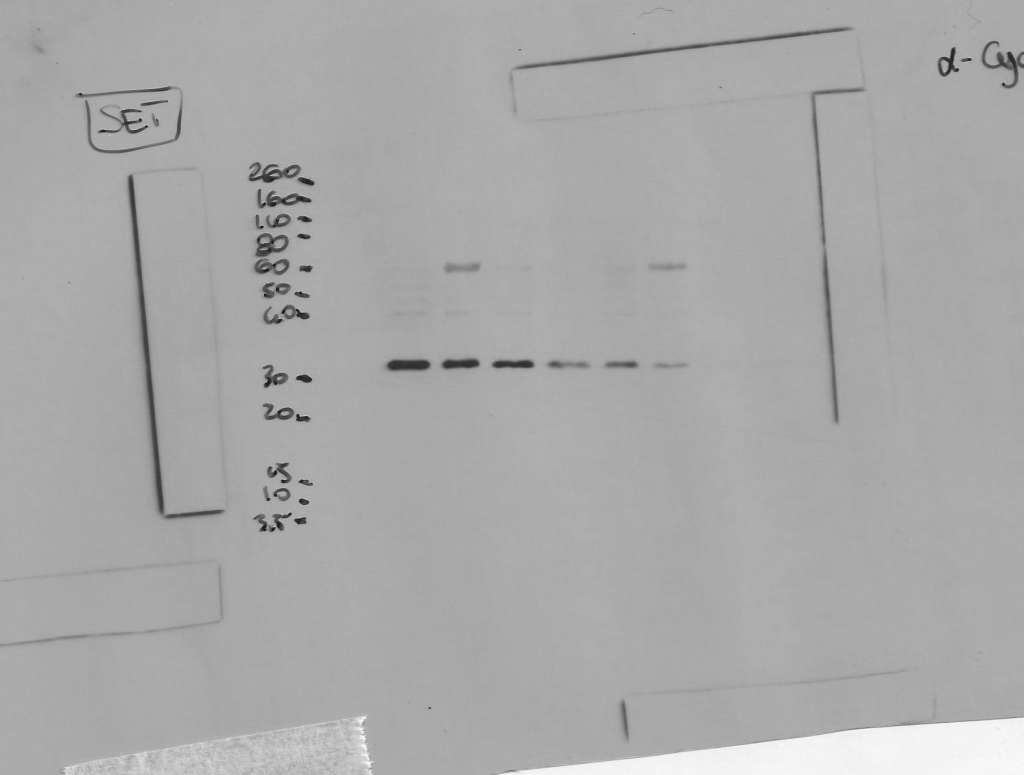

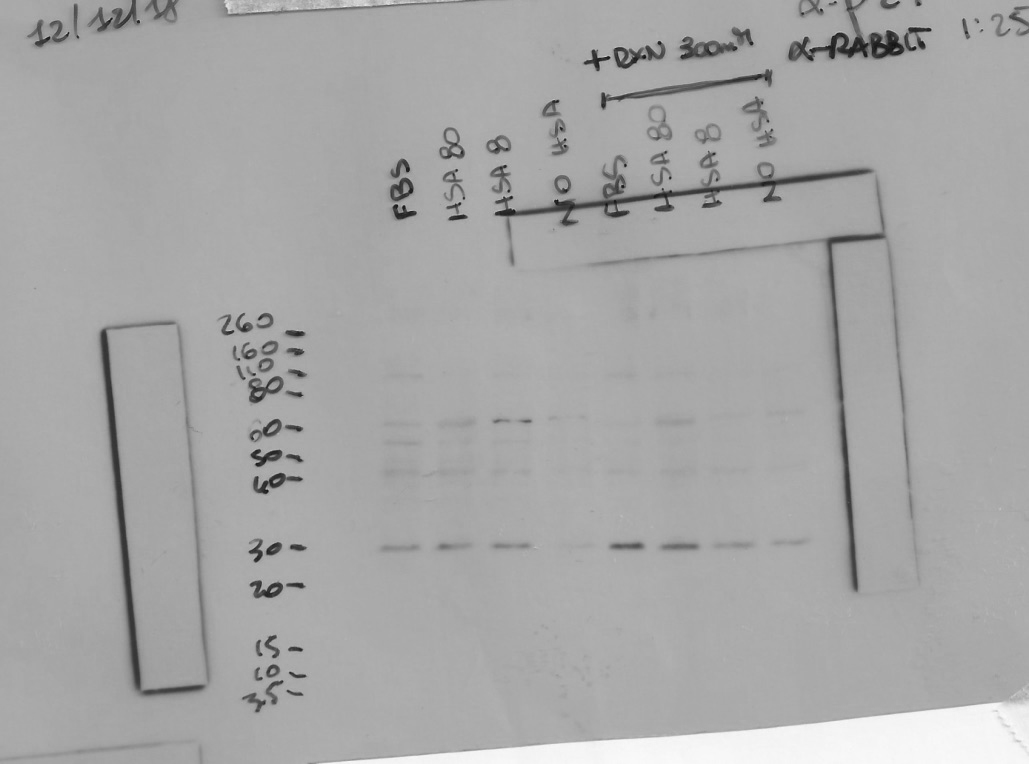

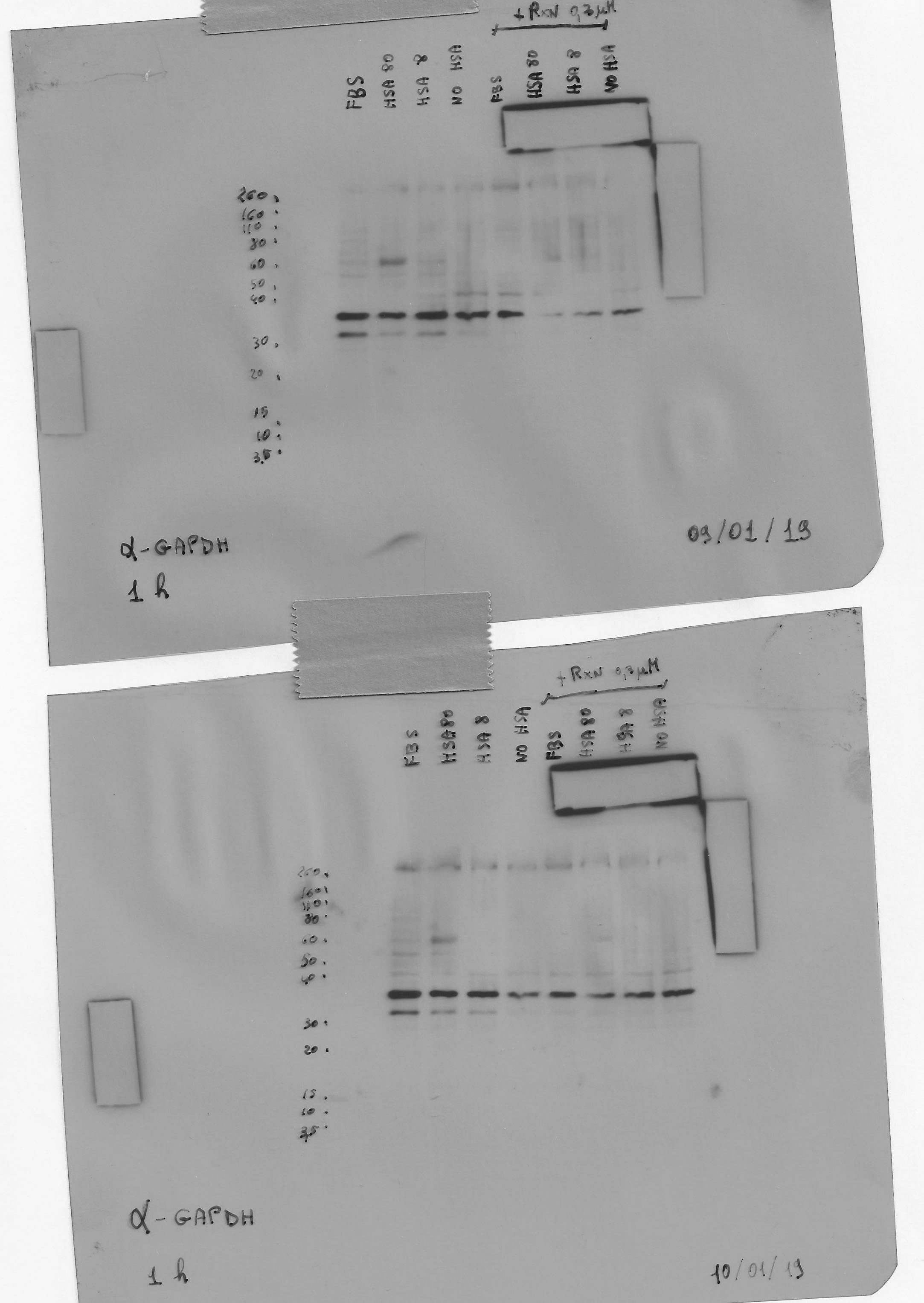


α -Cyclin D3 (33 kDa)

α -p27 (27 kDa)

Anti-GAPDH (37 kDa)

**S7**

Molecular weight ladder (kDa)

Molecular weight ladder (kDa)

1. FBS
2. HSA 80 μM
3. HSA 8 μM
4. HSA 0 μM
5. FBS + Rxn 0.2 μM
6. HSA 80 μM + Rxn 0.2 μM
7. HSA 8 μM + Rxn 0.2 μM
8. HSA 0 μM + Rxn 0.2 μM
9. Internal Control

1

2

3

4

5

6

7

8

9


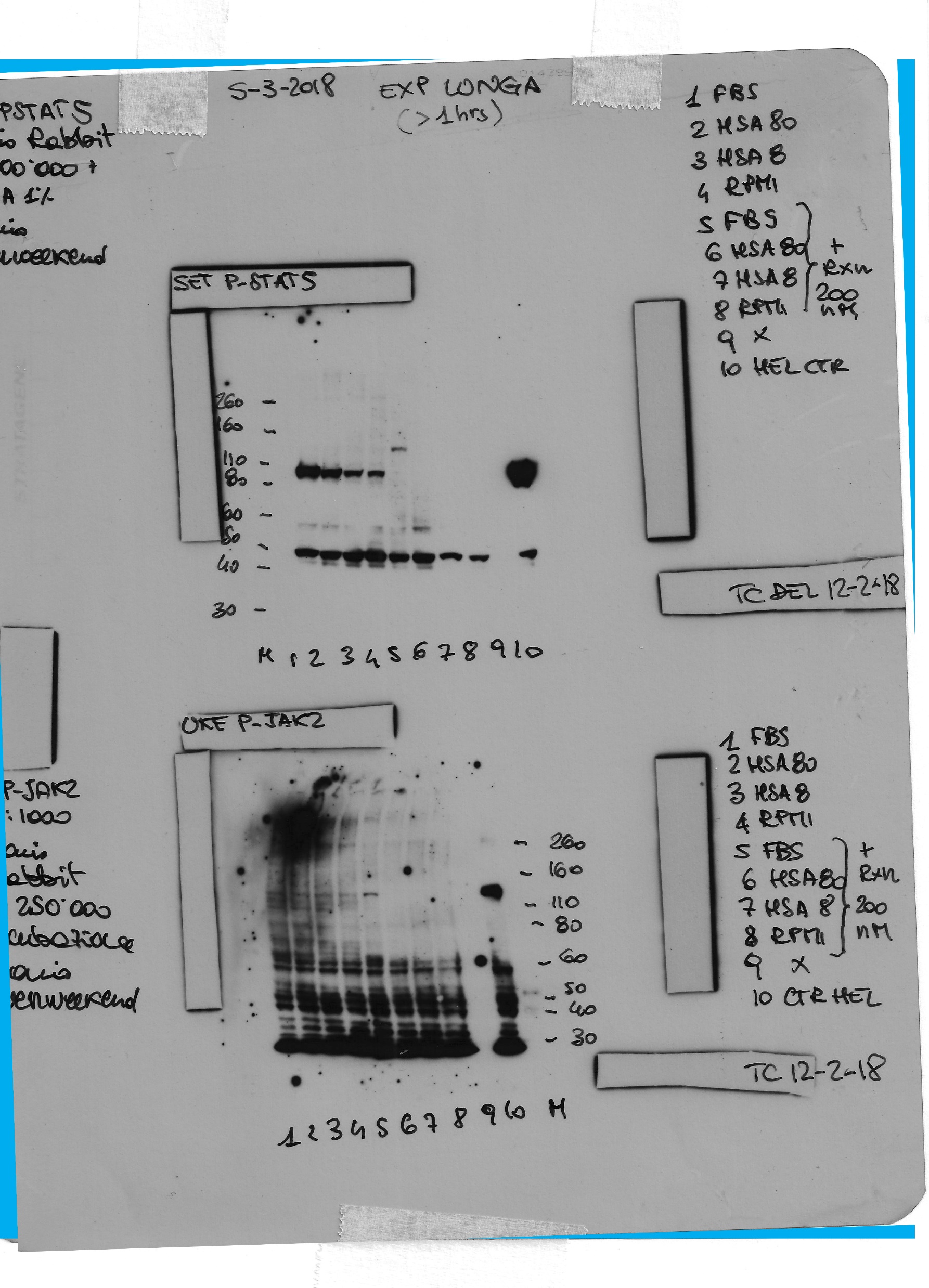

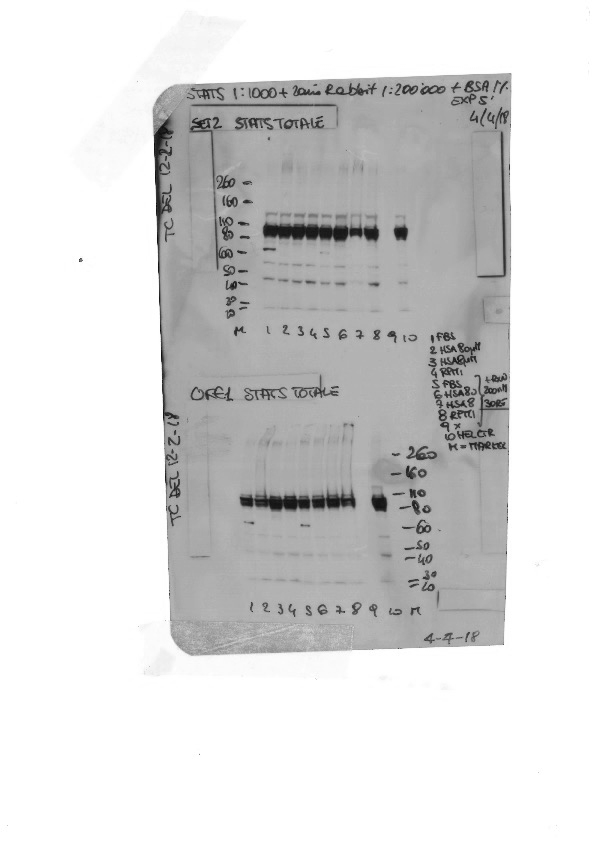


α -phospho-STAT5 (90 kDa)

α -STAT5 (90 kDa)

**S8**

**SET2 cells**

Molecular weight ladder (kDa)

Molecular weight ladder (kDa)

1. FBS
2. HSA 80 μM
3. HSA 8 μM
4. HSA 0 μM
5. FBS + Rxn 0.2 μM
6. HSA 80 μM + Rxn 0.2 μM
7. HSA 8 μM + Rxn 0.2 μM
8. HSA 0 μM + Rxn 0.2 μM
9. Internal Control

1

2

3

4

5

6

7

8

9


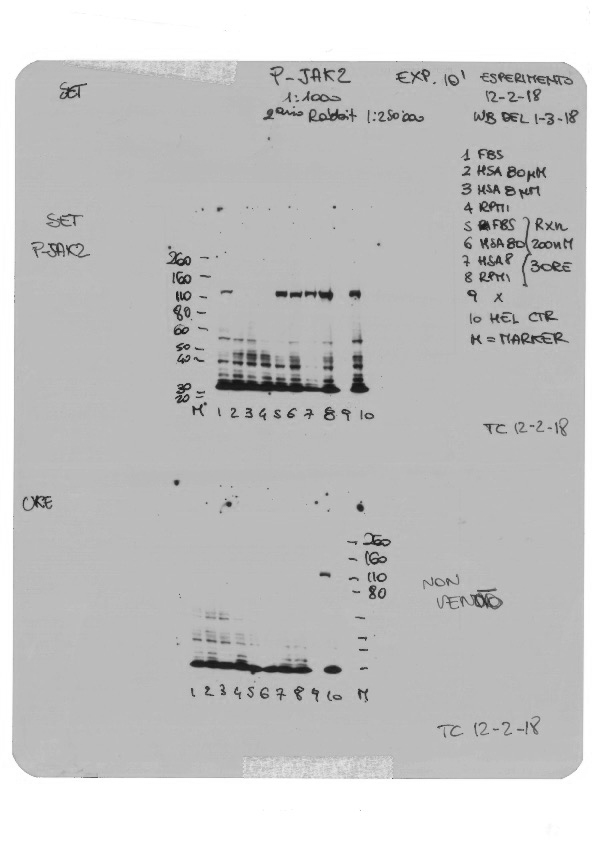

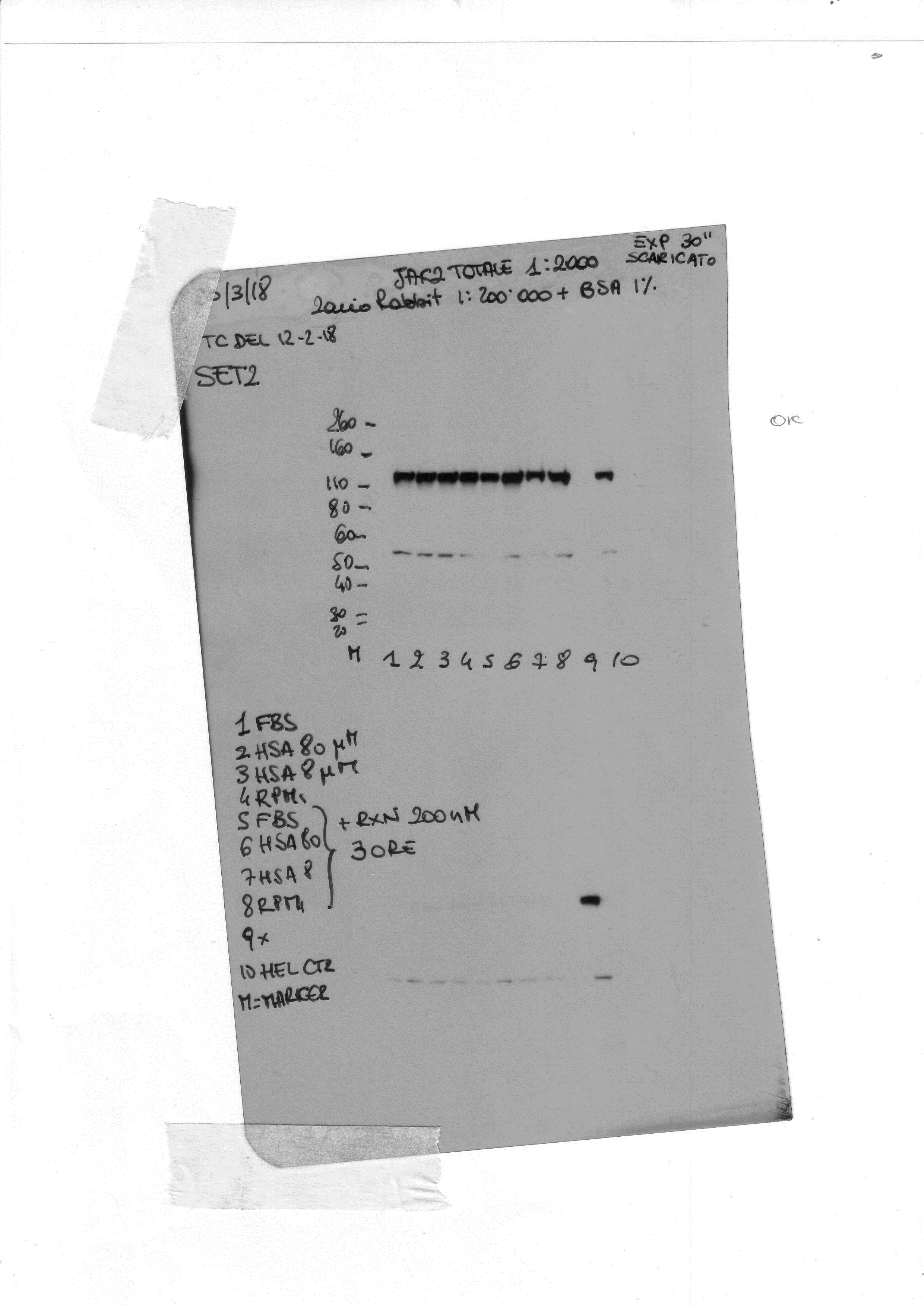


α -phospho-JAK2 (125 kDa)

α -JAK2 (125 kDa)

**S9**

**SET2 cells**

**Table S1.** Residues for which flexibility was allowed in docking simulations of ruxolitinib binding to ligand-free HSA.

| **FA binding site** | **Residues** |
| --- | --- |
| FA1 | Arg117, Leu182, Tyr161 |
| FA2 | Tyr150, Arg257, Ser287, Ala254 |
| FA3 | Ser342, Arg485, Arg348, Leu453 |
| FA4 | Tyr411, Ser489, Arg410, Leu457, Phe488, Val415, Leu460 |
| FA5 | Tyr 401, Lys525, Met548, Phe551 |
| FA6 | Arg209, Lys351, Ser480, Asp324, Glu354, Ala213 |
| FA7 | Lys199, Arg218, Arg222, His242, Val241 |
| FA8 | Lys195, Asp451, Ser454 |
| FA9 | Asp187, Lys432 |
